# *Callitriche cophocarpa* (water starwort) proteome under chromate stress: evidence for induction of a quinone reductase

**DOI:** 10.1007/s11356-017-1067-y

**Published:** 2018-01-13

**Authors:** Paweł Kaszycki, Aleksandra Dubicka-Lisowska, Joanna Augustynowicz, Barbara Piwowarczyk, Wojciech Wesołowski

**Affiliations:** 1 Unit of Biochemistry, Institute of Plant Biology and Biotechnology, Faculty of Biotechnology and Horticulture, University of Agriculture in Kraków, al. 29 Listopada 54, 31-425 Kraków, Poland; 2 Unit of Botany and Plant Physiology, Institute of Plant Biology and Biotechnology, Faculty of Biotechnology and Horticulture, University of Agriculture in Kraków, al. 29 Listopada 54, 31-425 Kraków, Poland; 3 Unit of Genetics, Plant Breeding and Seed Science, Institute of Plant Biology and Biotechnology, Faculty of Biotechnology and Horticulture, University of Agriculture in Kraków, al. 29 Listopada 54, 31-425 Kraków, Poland

**Keywords:** Macrophyte, Aquatic plants, Hexavalent chromium, Biological reduction, Phytoremediation, Oxidative stress, Quinone dehydrogenase

## Abstract

Chromate-induced physiological stress in a water-submerged macrophyte *Callitriche cophocarpa* Sendtn. (water starwort) was tested at the proteomic level. The oxidative stress status of the plant treated with 1 mM Cr(VI) for 3 days revealed stimulation of peroxidases whereas catalase and superoxide dismutase activities were similar to the control levels. Employing two-dimensional electrophoresis, comparative proteomics enabled to detect five differentiating proteins subjected to identification with mass spectrometry followed by an NCBI database search. Cr(VI) incubation led to induction of light harvesting chlorophyll *a*/*b* binding protein with a concomitant decrease of accumulation of ribulose bisphosphate carboxylase (RuBisCO). The main finding was, however, the identification of an NAD(P)H-dependent dehydrogenase FQR1, detectable only in Cr(VI)-treated plants. The FQR1 flavoenzyme is known to be responsive to oxidative stress and to act as a detoxification protein by protecting the cells against oxidative damage. It exhibits the in vitro quinone reductase activity and is capable of catalyzing two-electron transfer from NAD(P)H to several substrates, presumably including Cr(VI). The enhanced accumulation of FQR1 was chromate-specific since other stressful conditions, such as salt, temperature, and oxidative stresses, all failed to induce the protein. Zymographic analysis of chromate-treated *Callitriche* shoots showed a novel enzymatic protein band whose activity was attributed to the newly identified enzyme. We suggest that Cr(VI) phytoremediation with *C. cophocarpa* can be promoted by chromate reductase activity produced by the induced quinone oxidoreductase which might take part in Cr(VI) → Cr(III) bioreduction process and thus enable the plant to cope with the chromate-generated oxidative stress.

**Electronic supplementary material:**

The online version of this article (10.1007/s11356-017-1067-y) contains supplementary material, which is available to authorized users.

## Introduction

Anthropogenic pollution with Cr compounds has become a worldwide problem due to extensive use of this metal in a number of industrial applications and vehicular transport (Shadreck and Mugadza 2013; Singh et al. 2013; Zayed and Terry 2003). Chromium may appear at various oxidation states forming chemical agents of different toxicities, solubilities, and stabilities (Kotaś and Stasicka 2000; Zayed and Terry 2003), and among these, chromate (Cr(VI)) is the most hazardous to living organisms (Saha et al. 2011; Zhitkovich 2011). Chromate ion (CrO_4_^2−^) structurally resembles the sulfate anion (SO_4_^2−^) and therefore it becomes actively incorporated by cells mainly through non-specific sulfate transporter systems and in the lesser extent through HPO_4_^2−^ carriers (Cervantes et al. 2001; Prado et al. 2016; Singh et al. 2013). Then, inside cells, Cr(VI) as a strong oxidant interacts with cell constituents and undergoes rapid reduction due to both enzymatic and non-enzymatic reactions (Cervantes et al. 2001; Chandra and Kulshreshtha 2004; Shanker et al. 2005, 2009). Chromate bioreduction is in fact a gradual and complex route involving formation of Cr(V) and Cr(IV) intermediates responsible for generation of reactive oxygen species (ROS). In plants, many of these byproducts lead to adverse stress effects by causing severe physiological disorders and oxidative damage (Oliveira 2012; Panda and Choudhury 2005; Singh et al. 2013).

For the above reasons, Cr decontamination actions need to be undertaken. Among various methods implemented to date environmentally, bioremediation and especially phytoremediation appear as the most promising approaches (Parvaiz 2016). In biotechnological applications, plants capable of enhanced chromate uptake and accumulation as well as of its reduction to the less toxic forms are of particular interest since they bring possibilities for elaborating cheap, non-invasive, and efficient industrial-scale methods for soil reclamation and water cleanup (Prado et al. 2016; Yang et al. 2005; Zayed and Terry 2003). However, biological remediation of environmental contamination with chromium compounds involves complex and divergent processes (Chandra and Kulshreshtha 2004; Panda and Choudhury 2005; Shanker et al. 2005, 2009). In the case of higher plants, chromium stress negatively affects cellular metabolism at different functional levels triggering variant reactions that enable some species to adapt to this heavy metal, resist its action, or detoxify hazardous intermediates (Prado et al. 2016; Shanker et al. 2009; Zayed and Terry 2003).

Chromate detoxication and/or protective mechanisms in plants are complex, may involve multiple factors, and can become manifested at different organizational levels related to plant metabolism, physiology, development, or structure. For the above reasons, they are not yet fully explained and understood (see Prado et al. 2016 for a recent critical review). Moreover, these processes differ depending on the strategy used by particular species to cope with the metal toxicity. Based on these divergent strategies, plants can be categorized as Cr excluders, indicators, or accumulators (Dalvi and Bhalerao 2013). For the case of Cr-accumulating and Cr-tolerant plants, it is well evidenced that the chromate stress reaction involves induction of oxidative stress response systems. This is achieved by enhancing the activities of antioxidant enzymes (superoxide dismutase (SOD), catalase (CAT), peroxidase, glutathione reductase, ascorbate peroxidase, dehydroascorbate reductase, monodehydroascorbate reductase, as well as several others, Ovečka and Takáč 2014; Shanker et al. 2005; Singh et al. 2013) as well as by stimulating bioreduction of Cr(VI) to the final Cr(III) form considered more stable and less toxic (see discussion below). The described processes may, in turn, result in altered proteomic profiles whose detailed analysis is expected to broaden our knowledge on Cr bioconversion mechanisms and response strategies.

The mechanisms of chromate uptake, accumulation, and metabolism have been studied for terrestrial plants more thoroughly than for macrophytes (Shanker et al. 2005, 2009), although the latter group seems to be a better choice for phytoremediation purposes (Tel-Or and Forni 2011). This is because aquatic plants, especially the water-floating and submerged species, usually exhibit higher accumulation potential and have evolved distinct physiological and biochemical pathways due to their larger contact area with the surrounding water environment (Chandra and Kulshreshtha 2004). In consequence, the assimilative organs of macrophytes are affected directly by the solution and this interaction becomes particularly important in the case of heavy metal presence.

Several aquatic plants have been proposed as efficient phytoremediators of Cr contamination (Prado et al. 2016; Singh et al. 2013, and the references therein). Among these is the recently studied genus *Callitriche* that appears as a suitable candidate for environmental biotechnological use (Augustynowicz et al. 2014b; Favas et al. 2012, 2014). In particular, *Callitriche cophocarpa* Sendtn. (water starwort), a widespread species growing both in stagnant and running waters, was shown to reveal unusual response to chromate proving to be a potent Cr phytoremediator and accumulator (Augustynowicz et al. 2010, 2013a, 2013b, 2016). It exhibited enhanced capability of both Cr(VI) and Cr(III) phytoextraction from contaminated waters and extremely high capacity to bind Cr (3900 mg kg^−1^ for 5-day treatment at 1 mM Cr(VI)). Furthermore, it revealed extraordinary potential in terms of chromate accumulation rate (up to 1.8% of dry mass which makes it a Cr hyperaccumulator), intratissular bioreduction kinetics (a postulated detoxication pathway), and bioconcentration factor (BCF determined as 74 for a 5-day treatment of the shoots with 1 mM Cr(VI), the value close to that of commercially used sorbents). Upon incubation with 50-μM chromate, *C. cophocarpa* accumulated > 0.1% of Cr with no noticeable changes in the plant physiological status. All the above characteristics imply that the macrophyte has evolved efficient adaptive mechanisms against chromate stress and can respond to Cr(VI) treatment atypically.

So far, little has been done with regard to the studies of chromate-induced response of macrophytes at the level of detectable proteomes. Such research is of high interest, especially for the case of submerged plants that can interact with Cr compounds directly via their shoots. Therefore, besides particular applicability of *C. cophocarpa* for phytoremediation of aquatic systems, this species can serve as a good model to study chromium effect on cells and tissues. The main aim of the work is to reveal whether chromate treatment alters a protein profile of water starwort as detected with two-dimensional electrophoresis. Then, based on identification of the differentiating proteins with mass-spectrometry and bioinformatic tools, an attempt was made to establish whether the observed changes are specific to Cr(VI) and if they can account for the abovementioned unusual *C. cophocarpa* phytoremediation properties.

## Materials and methods

### Plant material

For the study, *C. cophocarpa* Sendtn. was grown under in vitro conditions. The source plant material had earlier been collected from its natural habitat: the Dłubnia river located in Southern Poland (N 50° 15′ 58″/E 19° 56′ 24.9″) during spring of 2012 (May). Healthy, undamaged shoots were rinsed for 10 min with a running, tap water. Next, the material was surface-sterilized in 70% ethanol for 25 s, followed by immersing in 0.5% aqueous solution of sodium hypochlorite for 2 min, and finally, rinsing five times with the sterile distilled water. Sterilized shoot fragments with apical buds (5–10-mm length) were placed in a liquid MS medium (Murashige and Skoog 1962) composed of macro- and microelements supplemented with vitamins and 1% sucrose. Every 6–8 weeks, the young shoots were transferred to a fresh medium. Plants were grown in a phytotron chamber (model FD 500, Biosell, Poland) under the following conditions: 16 h of photon flux density of 40 μmol m^−2^ s^−1^ and 8 h of darkness, at 22-°C day/18-°C night. Light spectrum and intensity were similar to that applied in previous studies (Augustynowicz et al. 2016) and were found sufficient for normal plant growth resembling the conditions typical of *Callitriche* natural environment.

### Plant incubation with chromate

Two grams of *C. cophocarpa* shoots was typically incubated for 72 h in 100 ml of MS basal salt medium supplemented with 1-mM chromate (CrO_4_^2−^) applied by dissolving potassium chromate K_2_CrO_4_ 10-mM stock solution directly into the plant medium (the chemical formula of potassium chromate, given as canonical SMILES is as follows: [O−][Cr](=O)(=O)[O−]·[K+]·[K+]; for detailed information see compound summary for CID 24597 in the PubChem Open Chemistry Database, https://pubchem.ncbi.nlm.nih.gov/compound/24597#section=Top; accession date: November 2017). Time, temperature, and light regimes of cultivation were as described above. Such conditions were established to cause cellular stress but were not lethal and upon removing Cr(VI) from the medium the plants fully recovered (see “Results”). In control experiments, the same amount of plant material was incubated in the MS medium. Morphological observations of *C. cophocarpa* shoots were carried out with a binocular microscope Nikon SMZ 1500.

### Other stress conditions

Temperature stress was introduced by 24-h incubation in the MS medium at 33 °C, then 48 h at control conditions. Salt stress was exerted by incubation in 150-mM NaCl for 24 h, then in MS medium for 48 h. Oxidative stress was applied either by 5-min incubation with 0.1-mM paraquat (methylviologen) or 5-min treatment with 1-mM hydrogen peroxide and then cultivation in the MS medium for 72 h. Note that all the applied stress-inducing factors had earlier been thoroughly tested at variant concentrations and treatment times. This enabled to obtain conditions providing considerable stress but not lethal to the plant (data not shown). All the incubations were performed in a phytotron chamber under the conditions described above. It was assumed that a 72-h incubation time was necessary for *Callitriche* to evolve the stress-like phenotype upon the tested factors, which was based on another study of *Lemna minor* L. time-dependent response to stress agents (Forni et al. 2012). After incubations, the plants were washed thrice in distilled water and kept frozen at − 20 °C.

### Preparation of protein samples for gel electrophoresis

Because of the relatively low protein concentration found for *Callitriche* tissues and very high amount of interfering compounds, especially phenolics and their glycosides (Augustynowicz et al. 2014a), the total plant material content for electrophoretic analyses was extracted using the method of phenol-SDS buffer extraction without sonication, elaborated by Chatterjee et al. (2012) with some necessary modifications. One gram of shoots was ground with liquid nitrogen in a mortar and extracted with 3 ml of homogenization buffer containing 30% sucrose, 2% SDS, 0.1-M Tris-HCl, 5% β-mercaptoethanol, and 1-mM PMSF (phenyl methyl sulfonyl fluoride), pH 8.0. Then, 3 ml of Tris-buffered phenol was added and the mixture vortexed for 15 min at 4 °C, then centrifuged at 8000 ×*g* at 4 °C for 15 min. The phenolic fraction was collected, 3 ml of a homogenization buffer added again, and the procedure of shaking and centrifugation was repeated. The phenolic phase was collected, centrifuged, and precipitated with 0.1-mM ammonium acetate in cold methanol at − 20 °C overnight. Then, the mixture was centrifuged at 10,000 ×*g* for 30 min at 4 °C and the pellet washed three times with 0.1-mM ammonium acetate in cold methanol and once with 80% cold acetone. The resultant precipitate was dried at room temperature and kept frozen at − 80 °C.

### SDS-PAGE electrophoresis

Protein pellets were solubilized and denatured with a loading buffer (0.5-M Tris-HCl, pH 6.8, 10% SDS, 10% β-mercaptoethanol, 20% glycerol, 0.05% bromophenol blue) at 100 °C for 5 min. SDS-PAGE was performed according to Laemmli (1970) using a BioRad MiniProtean System, applying 4% stacking and 10% separating polyacrylamide gels at 20-mA per gel. The total of 30 μg protein was loaded per well. After electrophoresis, the gels were stained with a Coomasie Brillant Blue R250 (Sigma). For calibration of molecular mass a BlueEye Prestained Protein Marker (Sigma) set was used. Protein concentration in all extracts and samples was determined using a Bradford (1976) method with bovine serum albumin (BSA) as a standard.

### 2DE electrophoresis

For protein isoelectrofocusing (IEF, the first dimension), the protein pellet was solubilized in a rehydration buffer (7-M urea, 2-M thiourea, 2% CHAPS, 0.002% bromophenol blue, 20-mM DTT, 1% ampholyte buffer (BioLyte, BioRad)) to a final volume of 150 μl and loaded onto 7-cm IPG strips (BioRad Ready Strip) of the pI range 3–10, and then, in an independent run, at pI range 5–8. Passive rehydration was carried out for 12 h at 20 °C and the isoelectric focusing was performed at 20 °C (Protean IEF Cell, first step 250 V for 20 min, second step 4000 V for 120 min, third step 4000 V, 10,000 V h). Prior to the SDS-PAGE, the IPG strips were equilibrated for 10 min in buffer I containing 1% DTT, 6-M urea, 75-mM Tris-HCl, pH 8.8, 30% glycerol, and 2% SDS and then, in the second step, for 10 min in buffer II consisting of 2.5% iodoacetamide, 6-M urea, 75-mM Tris HCl, pH 8.8, 30% glycerol, and 2% SDS. The SDS-PAGE (the second dimension) was performed according to the protocol described in the section above using a BioRad Protean II xi Cell 16 × 16-cm slab unit. In order to achieve maximum reproducibility of spot patterns for proteome comparison, a pair of IPG strips, obtained upon IEF of either control or Cr-treatment experiment, were placed side-by-side onto one 16-cm polyacrylamide gel and then overlaid with low melting-point agarose (ReadyPrep overlay agarose, BioRad). Protein separation was carried out at 20 mA per gel for about 6 h. After electrophoresis, the gels were silver-stained as described by Jungblut and Seifert (1990), scanned, and digitalized (DNr Bio-Imaging Systems MiniBis Pro, Israel, equipped with a 16-bit, 1.3 Mpix CCD camera). Then, the resultant proteome maps were matched to identify differentiating spots. Spot detection and quantification were done automatically employing either the Vision Works 2D Lite (UVP) software or Melanie 7.0 (Genebio), followed by manual verification. The amount of protein in each analyzed spot was calculated as a spot pixel density (grayscale) after subtracting gel background value. Then, to compensate for variability of gel staining, protein abundance was given as a relative density upon normalization procedure based on the densities of four selected spot marks representing invariant proteins that appeared on all the tested gels. For each set of differentiating spots in replicated gels, standard deviation of the fold change was calculated.

Three *Callitriche* extracts obtained upon independent physiological experiments were used for proteome mapping. Then, electrophoretic protein profiling of each set of extracts (Cr(VI) treatment and control) was done twice, using IPG strips of two pH ranges: 3–10 and 5–8. Spot pattern differences were examined for all the replicate gel pairs. In order to properly select only the differentially abundant proteins, we made sure that the candidate spots showed accumulation changes in all of the gel replicates.

For subsequent MS/MS analyses, spot excision was made using the 2DE gels stained without adding glutaraldehyde to the sensitizing solution in order to improve protein identification. Also, to reduce the number of overlapping proteins, the IEF step was performed at pH ranging from 5 to 8.

### MS/MS protein identification

Protein identification was done at the Institute of Biochemistry and Biophysics of the Polish Academy of Sciences, Warsaw, Poland. The differentiating spots were excised manually and placed in Eppendorf tubes. Then, the gel pieces were dried with acetonitrile and subjected to reduction with 10-mM DTT in 100-mM NH_4_HCO_3_ for 30 min at 57 °C. Cysteine residues were alkylated with 0.5-M iodoacetamide in 100-mM NH_4_HCO_3_ (45 min in a darkroom at room temperature) and the proteins were digested overnight with 10-ng/μl trypsin in 25-mM NH_4_HCO_3_ (Promega) at 37 °C. The resultant peptide samples were concentrated and desalted on a RP-C18 precolumn (Waters). Further peptide separation was achieved on a nanoultra performance liquid chromatography (UPLC) RP-C18 column (Waters, BEH130 C18 column, 75-μm i.d., 250-mm length) of a nanoACQUITY UPLC system, using a 45 min linear acetonitrile gradient. The column outlet was directly coupled to the electrospray ionization (ESI, voltage of 1.5 kV) ion source of the Orbitrap Velos type mass spectrometer (Thermo), working in the regime of data dependent MS to MS/MS switch with HCD type peptide fragmentation. A blank run to ensure that there was no cross contamination from previous samples preceded each analysis. Raw data files were preprocessed with Mascot Distiller software (version 2.5, MatrixScience). The obtained peptide masses and fragmentation spectra were matched to the National Center Biotechnology Information (NCBI) non-redundant database no. 20150115 (57,412,064 sequences; 20,591,031,683 residues), with a *Viridiplantae* filter using the Mascot search engine (Mascot Server v. 2.4.1, MatrixScience) and the probability-based algorithm. The following search parameters were applied: enzyme specificity set to trypsin, peptide mass tolerance to ± 30 ppm, and fragment mass tolerance to ± 0.1 Da. The protein mass was left as unrestricted and mass values as monoisotopic with one missed cleavage being allowed. Alkylation of cysteine by carbamidomethylation was set as fixed and the oxidation of methionine as a variable modification. Multidimensional protein identification technology-type (MudPIT-type) and/or the highest number of peptide sequences were selected. Mascot peptide ion scores served as bases for ranking protein hits. Each ion score was expressed as – 10 · log(*P*), where *P* is the probability that the observed match was a random event. Individual ions scores ≥ 43 indicated identity or extensive homology (*p* < 0.05). The highest scores in the NCBI database search were matched to each analyzed spot.

### Preparation of protein extracts for enzymatic analyses

To obtain native protein extracts for zymographic analysis of quinone reductase (QR), 0.8 g of shoots was frozen in liquid nitrogen, ground in a mortar, and suspended in 1.1 ml of homogenization buffer containing 100-mM Tris-acetate, pH 8.0, 100-mM potassium acetate, pH 8.0, 2-mM EDTA, 5-mM DTT, 250-mM sodium ascorbate, and 10% *v*/*v* glycerol (Laskowski et al. 2002). The samples were centrifuged at 10,000 ×*g*, 4 °C for 15 min, and the supernatant was collected. For activity assays of antioxidant enzymes, 0.8 g of frozen shoots was ground in a mortar and extracted with 2 ml of 50-mM Tris-HCl buffer, pH 7.0, containing 1 mM of PMSF. Protein extracts were centrifuged as above.

### Zymographic analysis of quinone reductase (QR)

For zymographic QR assay, a native-PAGE electrophoresis was performed at non-denaturing conditions using 10% separating gel and a 4% stacking gel. Samples were mixed with a loading buffer containing 0.5-mM Tris-HCl, pH 6.8, 20% glycerol, and 0.05% bromophenol blue and then 13 μg of protein was loaded per well. Electrophoresis was carried out at 50 V for 20 min and then at 75 V per gel (Mini Protean System, BioRad). The enzymatic staining technique was elaborated based on a QR assay described by Prochaska and Santamaria (1988) and Sharma et al. (1996) with some modifications. The reaction mixture contained 4.5 ml of 0.5-M Tris, 600-μl Tween-20, 60 mg of BSA, 27 mg of MTT (3-(4,5-dimethylthiazol-2-yl)-2,5-diphenyl tetrazolium bromide), and 90 μl of 50-mM menadione substrate. The reaction was launched with 54 μl of 50-mM NADPH. The resultant gels were digitalized after 60 min of staining.

### Assays of enzymatic markers of oxidative stress

Catalase (CAT) activity was measured with a modified spectrophotometric method of Aebi (1984) by a decrease of H_2_O_2_ as observed at 240 nm in a reaction mixture containing 1 ml of 54-mM solution of hydrogen peroxide in 50-mM potassium phosphate buffer, pH 7.0, and 1.8 ml of the buffer. The reaction was initiated with the addition of 200 μl of a sample and the absorbance decrease was measured at 240 nm after 1 min. Superoxide dismutase (SOD) activity was determined according to the method of Beauchamp and Fridovich (1971) with some modifications. The reagent mix was prepared by mixing of 2.18 ml of 100-mM potassium phosphate buffer, pH 7.8, 0.4 ml of 1.5-mM NBT (nitro blue tetrazolium), 0.2 ml of 55-mM methionine, 0.2 ml of 0.12-mM riboflavin, and 20 μl of a sample. The reaction solution was illuminated with a 36-W fluorescent lamp for 15 min. A decrease in absorbance was measured spectrophotometrically at the wavelength of 560 nm. Peroxidases (total activity) were assayed according to Lück (1963). A 2.98-ml volume of 50-mM phosphate buffer, pH 6.2, was mixed with 0.1 ml of 1% p-phenylenediamine and 0.1 ml of 0.1% H_2_O_2_. The reaction was started with the addition of 20 μl of the 10-times diluted sample. The absorbance at 485 nm was measured after 1 and 2 min of incubation. Specific activities of all the above enzymes were expressed as numbers of activity units per mg protein. All the assays were done in triplicates. Statistical evaluation was performed with the one-way ANOVA variance analysis. Statistical differences between mean values were verified with a Tukey post hoc test (*α* = 0.05 and *n* = 3, MATHLAB 2016a statistical software module).

### Reagents

All the chemicals and reagents were of analytical or electrophoretic grades. Nanopure water, IPG strips, agarose, and ampholyte solution were from BioRad. Sucrose, potassium acetate, phosphate, sodium ascorbate, sodium chloride, and hydrogen peroxide were obtained from POCh Gliwice, Poland. The MS medium was purchased from Duchefa. Methylviologen (paraquat) and NBT were from MP Biomedicals. All other chemicals and enzymes were purchased from Sigma-Aldrich.

## Results

**Fig. 1:**
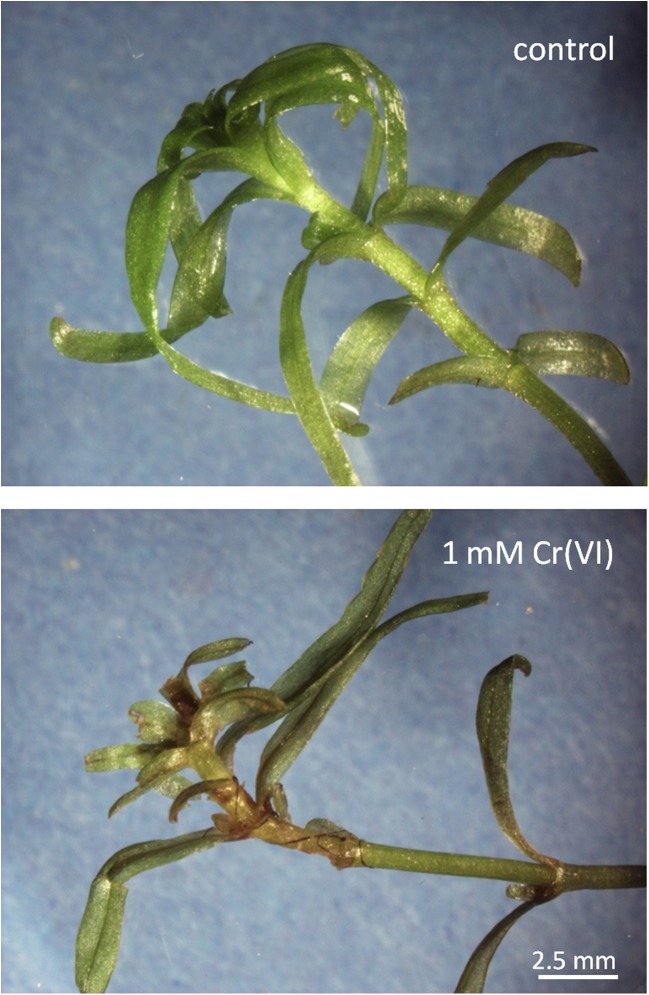
Representative photographs of *C. cophocarpa* apex shoots of control plants and the ones treated for 72 h with 1-mM chromate

In order to study the proteomic response of *C. cophocarpa* to Cr(VI), the plants were incubated for 72 h with 1-mM potassium chromate, which is at conditions evaluated experimentally to be sublethal. In independent experiments of this study, it was established that the treatment caused physiological stress and led to morphological toxicity symptoms covering chlorosis and necrosis of shoots, shortening of apical internodes, and hampered development of apical buds (Fig. 1). Importantly, these effects were reversible since after washing out the Cr(VI) solution and further cultivating in the MS medium, full recovery was achieved within several days with no manifestations of any disorders.

**Fig. 2:**
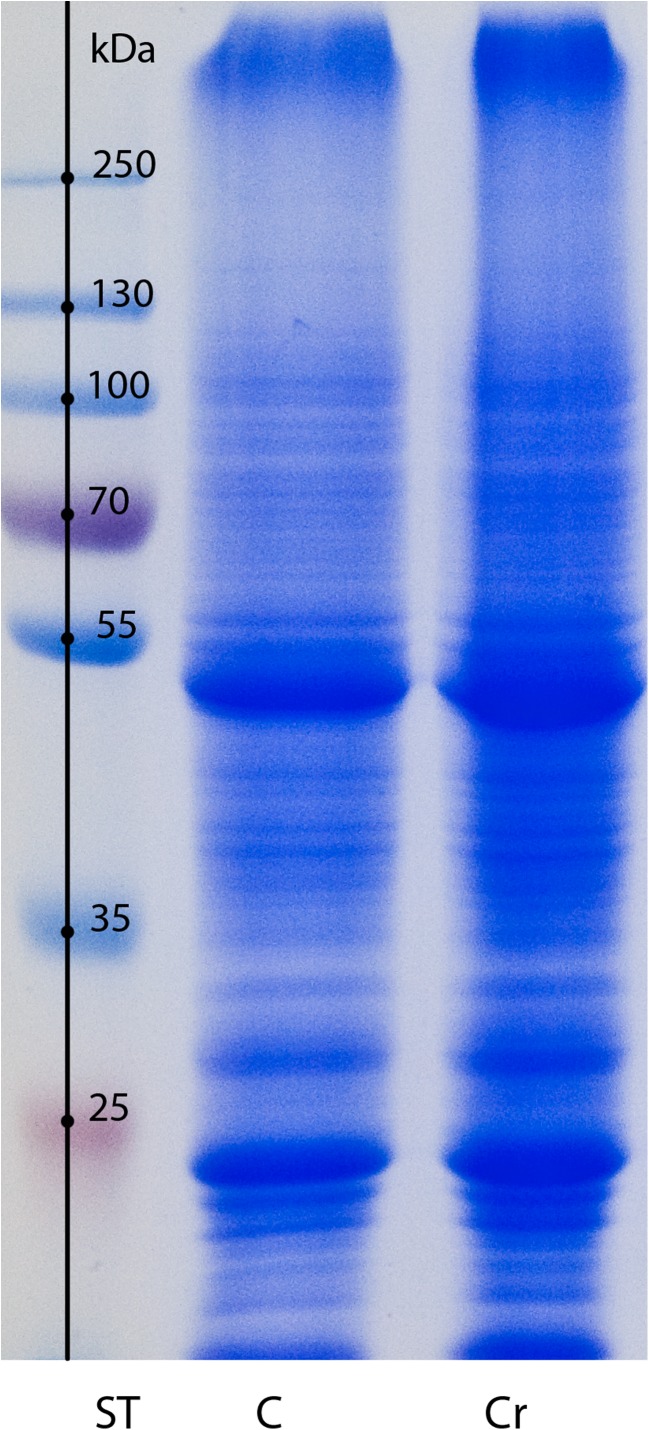
SDS-PAGE protein profiles of shoot extracts of *Callitriche cophocarpa* treated with 1-mM chromate for 72 h (Cr). C, control (untreated plant); ST, protein standard markers

**Fig. 3:**
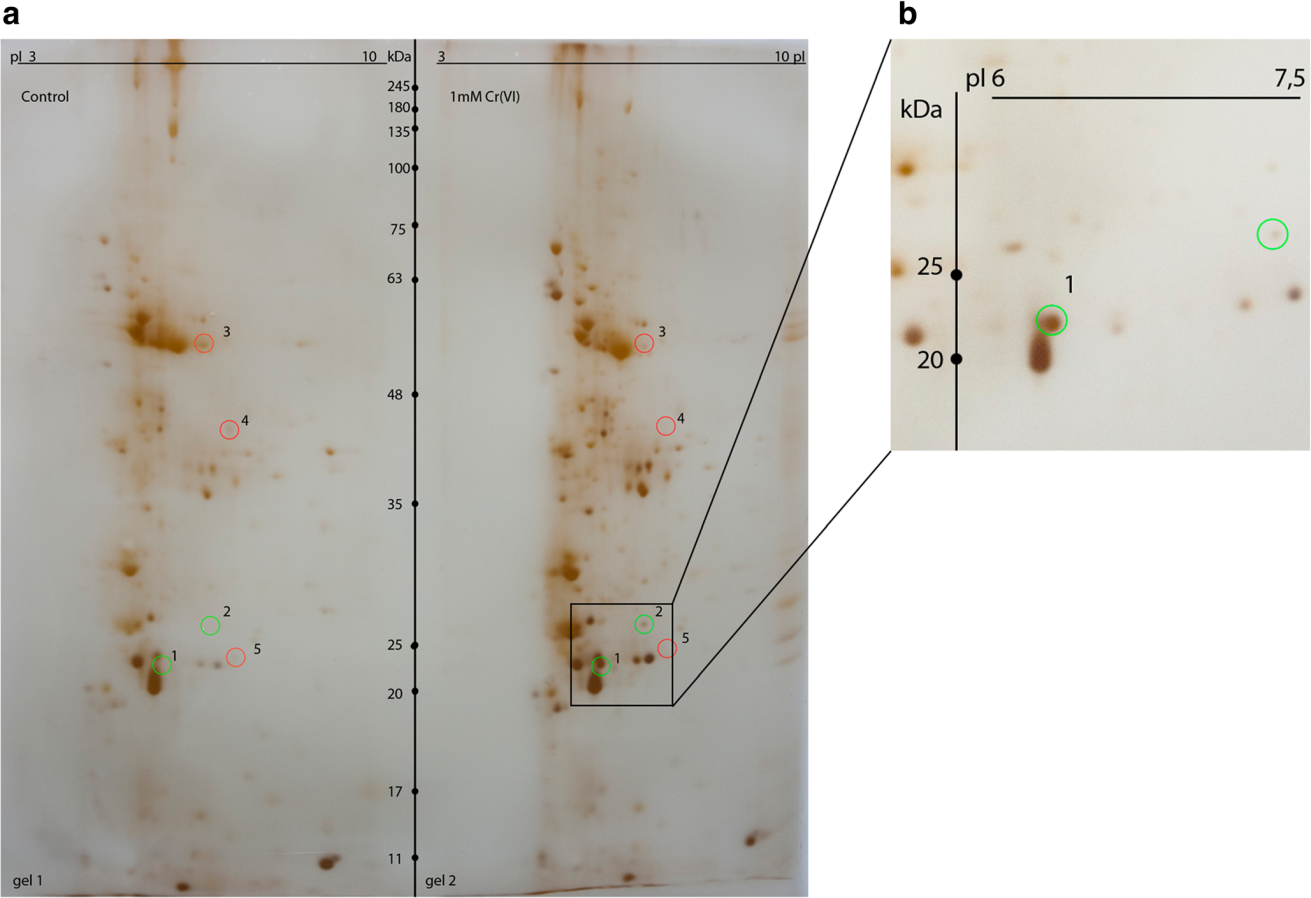
2DE proteome mapping of *C. cophocarpa* shoots after treatment with 1-mM potassium chromate for 72 h. **a** Isoelectric focusing at pI range 3–10. **b** 2DE gel fragment obtained with the pI range of 5–8 to extend the marked area of the gel (**a**). Differentiating spots are numbered consecutively and marked with circles; spot nos. 1 and 2 represent the Cr(VI)-induced proteins (green circles), nos. 3–5 indicate the down-regulated proteins (red circles)

Preliminary comparative protein profiling upon chromate treatment was carried out with the SDS-PAGE. The electrophoretic paths show no detectable differences in terms of protein patterns (Fig. 2). Approximately 45 bands can be detected, each revealing similar protein abundances for both control conditions and the Cr(VI)-treated plants. To obtain protein maps with enhanced resolution and protein detectability, two-dimensional electrophoresis was employed with a silver-staining technique. The resultant protein profiles, as exemplified in Fig. 3a for IPG strips ranging from pH 3 to 10, reveal five repetitive spot differences indicating proteins with altered accumulation. The spots have been numbered consecutively and they represent the proteins induced de novo (1, 2, marked by green circles) as well as the ones down-regulated (3, 4, 5, red circles) upon chromate treatment. The most pronounced difference can be observed for the spot no.1 which was strongly induced only by Cr(VI) presence and was never detected in the control experiment. That is why we decided to show the surrounding region (marked by a square in Fig. 3a) in a more detail that is using the IPG strip with the zoomed pH range of 5–8. The region of interest is presented in Fig. 3b. The applied narrower pI range allowed much higher resolution, prevented spot overlapping, and enabled to excise distinct spots, now well separated from the neighboring proteins. The spots of Fig. 3, qualified as the ones representing differentiating proteins, were excised from gels and subjected to MS/MS sequencing followed by bioinformatics-based identification. The results are given in Table 1.

**Table 1:** Differentiating proteins in proteomes of *Callitriche cophocarpa* treated with chromate (1 mM for 72 h) as compared to the control conditions (in vitro incubation). MS/MS protein identification was followed by bioinformatic analysis using NCBI databases confined to *Viridiplantae*

Spot	NCBI accession number	Score^a^	Protein name (species)	Number of matching peptides	Sequence coverage (%)	Molecular mass (kDa)		Isoelectric point (pI)		Expression change (fold change^d^)
						Theoretical^b^	Observed^c^	Theoretical^b^	Observed^c^	
1	XP_002283286	358	NAD(P)H dehydrogenase (quinone) FQR1 (*Vitis vinifera*)	8	21.2	21.7	21.5	5.8	6.3	Induced
2	CAA48410	219	Light harvesting chlorophyll *a*/*b* binding protein (*Hedera helix*)	3	9.3	20.8	24.2	4.8	7.3	Induced
3	AAF62288	1951	Ribulose 1,5-bisphosphate carboxylase (*Callitriche hamulata*)	25	46.1	50.1	58.0	6.3	6.7	0.8 ± 0.1
4	Q05554	368	Ribulose bisphosphate carboxylase large chain (*Antirrhinum majus*)	5	13.0	50.2	44.8	6.1	7.6	0.5 ± 0.2
5	CBG76811	276	Pollen allergen Sec c 5 (*Secale cereale*)	7	30.8	29.8	22.3	6.0	7.7	0.3 ± 0.1

^a^Given by Mascot

^b^Calculated from the respective sequence record

^c^Observed in the SDS-PAGE gel

^d^Fold change given as an averaged ratio of normalized spot densities as observed in treated and control samples ± standard deviations. The term “induced” indicates proteins detected exclusively upon Cr(VI) treatment

The most important observation is high-ranked identification of the induced protein with a *M*_W_ = 21.5 kDa and pI = 6.3 (the observed values, spot no. 1), found to be closely related to the FQR1 dehydrogenase from *Vitis vinifera* L.. This enzyme is a flavin mononucleotide-binding, NAD(P)H-dependent quinone dehydrogenase that exhibits the in vitro and presumably also in vivo activity of a quinone reductase (Laskowski et al. 2002). Among the other well-scored matches was the spot no. 2 with *M*_W_ = 24.2 kDa and pI = 7.3, identified as the light harvesting chlorophyll *a*/*b* binding protein (LHCB) from *Hedera helix* L. This was the second only protein found to be induced upon chromate stress. Spots no. 3 (the observed values of *M*_W_ = 58.0 kDa, pI = 6.7) and 4 (*M*_W_ = 44.8 kDa, pI = 7.6) represent down-regulated proteins identified as large RuBisCO subunits from *Callitriche hamulata* Kütz. and *Antirrhinum majus* L., respectively.

Note that for the case of spot no. 4, the initial, randomly made peptide scoring yielded unreliable match of a “hypothetical protein PRUPE_ppa008516mg from *Prunus persica* (L.) Batsch.” However, both the protein taxonomic status (*Prunus* genus distant from *Callitriche*) and discrepancies between the theoretical PRUPE molecular mass and pI values (*M*_W_ = 36 kDa and pI = 9.25) vs. the observed parameters (44 kDa and 6.13, respectively) gave strong reasons to reject this match. Moreover, PRUPE_ppa008516mg scored very similar to the second-in-order protein match of RuBisCO large chain of *A. majus* (Mascot scores of 369 compared to 368, respectively). In addition, the latter result was characterized by a larger number of matching peptides and higher sequence coverage (see Table 1 and Supplementary Table 1) and was further supported by several other matches with similar Mascot scores indicating RuBisCO from other *Callitriche* species (*C. brutia* Petagna, *C. cribrosa* Schotsman, *C. hamulata* Kütz., *C. platycarpa* Kütz., the results unshown). For the above reasons, the spot no. 4 was finally accepted to represent the RuBisCO large chain protein. For the spot 5, the down-regulated protein identification as a “pollen allergen” of *Secale cereale* L. is very difficult to interpret and suggests that it is an accidental match caused by a broad search through the huge database of *Viridiplantae*.

**Fig. 4:**
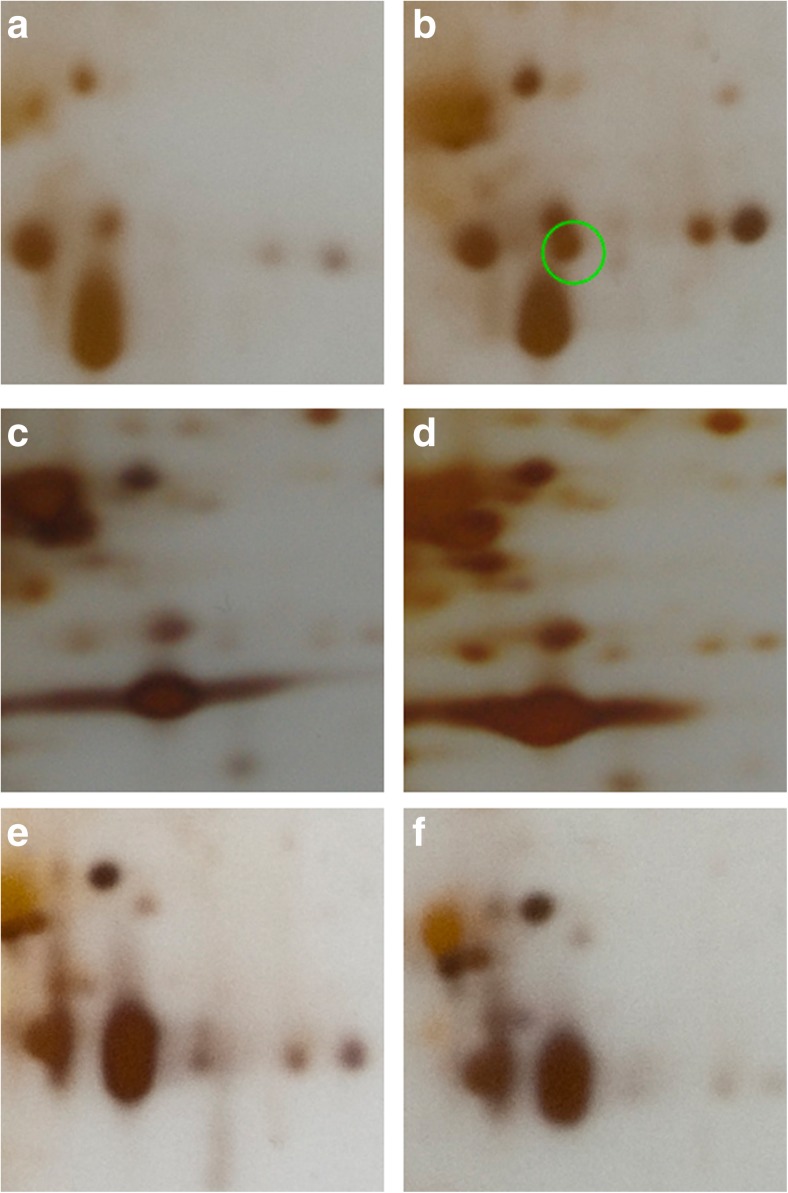
2DE gel fragments revealing *C. cophocarpa* protein profiles within the area of chromate-induced proteins 1 and 2 (FQR1 and LHCB, respectively), obtained for different stressors: **a** control (no stress applied), **b** Cr(VI) treatment, **c** paraquat oxidative stress, **d** hydrogen peroxide oxidative stress, **e** salt stress, **f** temperature stress

The chromate treatment proteomic data were compared with 2D protein profiles of *C. cophocarpa* shoots subjected to other stressors such as paraquat, hydrogen peroxide, sodium chloride, and elevated temperature. At this stage of the study, we focused on the gel region particularly affected by Cr(VI) presence, where the most prominent induction of proteins occurred, that is FQR1 and LHCB (the respective spots 1 and 2). The resultant fragments of protein maps are shown in Fig. 4c–f. Although they reveal some stress-specific differences when compared to the control (Fig. 4a), FQR1 and LHCB were never detected except for the Cr(VI) stress (Fig. 4b). This result proves that both proteins of interest were induced specifically by the presence of chromate.

**Fig. 5:**
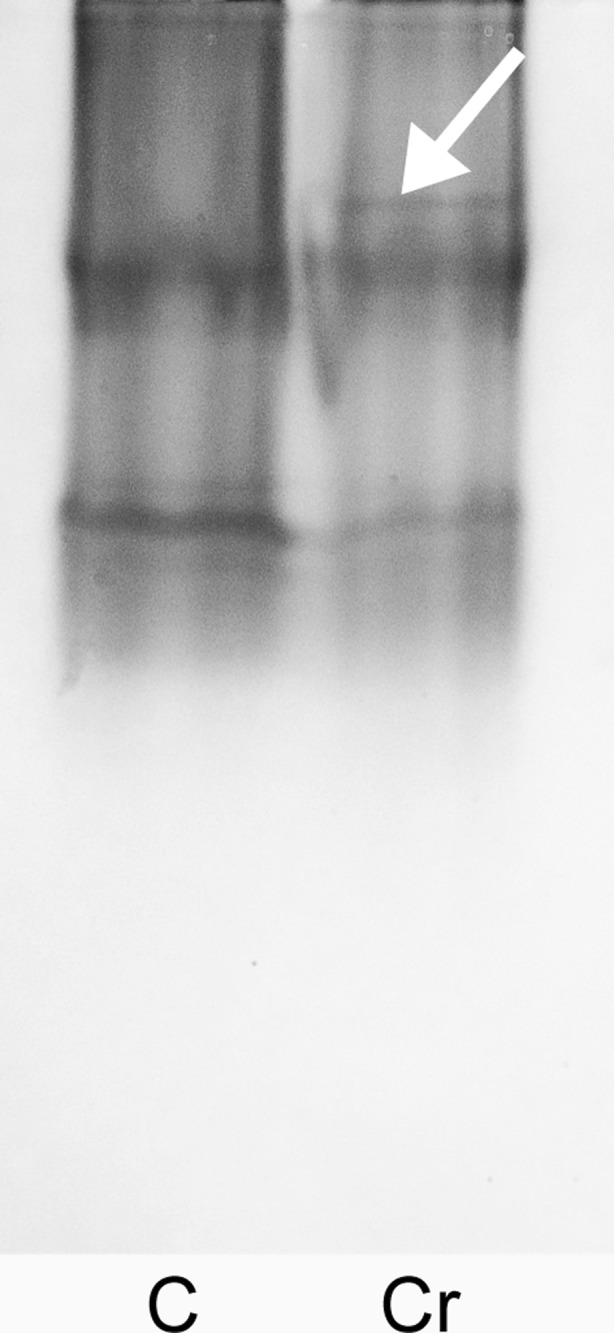
Zymographic analysis of shoot protein extracts of *Callitriche cophocarpa* treated with 1-mM Cr(VI) for 72 h. Enzymatic staining with a menadione substrate was done to reveal production of a novel quinone reductase activity as indicated by an arrow. C, control

In order to check for the QR activity of the chromate-induced FQR1 protein, extracts from *Callitriche* shoots preincubated with 1 mM Cr(VI) were tested zymographically with the menadione substrate. Figure 5 shows zymograms of native-PAGE gels obtained for the control (path C) and Cr(VI)-treated plants (path Cr). There are two major and two minor bands visible in both paths, and they possibly reflect some unspecific menadione-reducing reactions resulting from high content of phenols (see “Discussion”). However, an additional zymographic band appeared only within the Cr path (indicated with an arrow), thus providing evidence of a newly produced QR activity occurring in the Cr(VI)-treated plants.

**Fig. 6:**
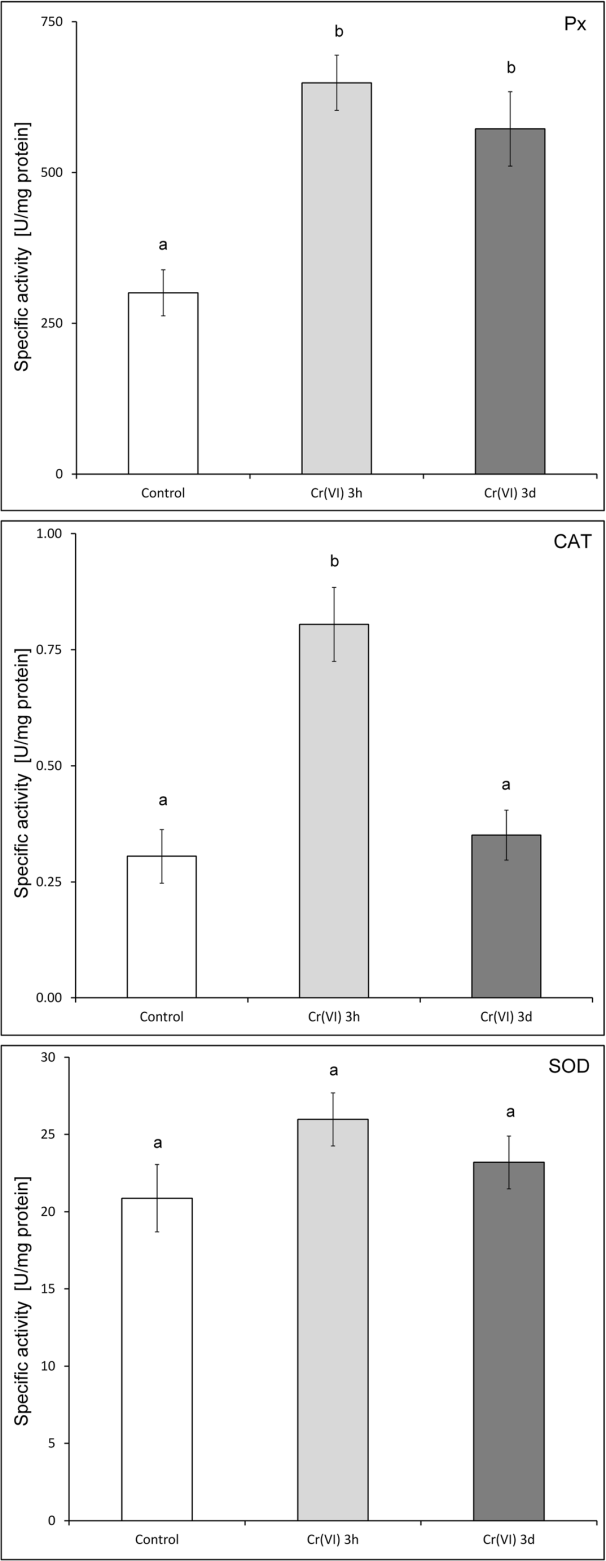
Specific activities of oxidative stress enzyme markers: Px, peroxidase; CAT, catalase; SOD, superoxide dismutase, obtained for shoot extracts of *Callitriche cophocarpa* subjected to chromate treatment for 3 (light-gray bars) and 72 h (3 days, dark-gray bars). White bars: control experiment (untreated shoots). The mean values of particular enzyme activities (*n* = 3 ± SD) marked with different letters are significantly different for *p* ˂ 0.05

The oxidative stress status of Cr(VI)-treated *C. cophocarpa* was evaluated based on activity analyses of selected enzymes known to be responsive to such stress that is peroxidases (Px), CAT, and SOD. The measurements were carried out at two time intervals, which is directly after treatment (3 h) and after 3-day incubation with a 1-mM chromate solution. The results were then compared to the values determined for control (untreated) plants. In Fig. 6, it can be clearly seen that Cr(VI) treatment caused over twofold induction of peroxidase(s) activity as measured after 3 h (from 301 ± 38 U/mg protein to 649 ± 45.60 U/mg) and the elevated value was kept after 3-day incubation (572 ± 61.77 U/mg). For CAT, in short-term (3 h) incubation, a strong activity increase was observed (0.31 ± 0.06 to 0.81 ± 0.08 U/mg). We note that this effect was very similar to the one caused by 15-min stressing of *Callitriche* cells directly with hydrogen peroxide (0.79 ± 0.02 U/mg, data not presented). However, the chromate effect on CAT activity was diminished upon long-term incubation and after 3 days, the resultant value was back at the level of control (0.35 ± 0.05 U/mg). In the case of SOD, no statistically significant activity changes were recorded for either 3-h or 3-day treatment with Cr(VI).

## Discussion

Abiotic stress is known to alter proteomic patterns in many plants (Jorrín-Novo et al. 2009). Comparative proteomics analyses can be a source of important novel information on the nature of stress tolerance and adaptation mechanisms and may suggest occurrence of newly induced biochemical pathways (Ahsan et al. 2009; Kosová et al. 2011; Timperio et al. 2008). For studies of heavy metal interactions with assimilative plant organs, aquatic plants are regarded as favorable models. Macrophytes are also preferable candidates for efficient phytoremediation in aqueous environments (Parvaiz 2016).

So far, many scientific contributions have been published, in which heavy metals were shown to affect plant proteomes (for reviews see DalCorso et al. 2013; Kosová et al. 2011; Ovečka and Takáč 2014; Visioli and Marmiroli 2013). However, the available proteomic reference data regarding chromium effect on plants is relatively poor and deal predominately with terrestrial plant models (Cvjetko et al. 2014; Hossain and Komatsu 2013; Vannini et al. 2011; Yildiz and Terzi 2016; Zemleduch-Barylska and Lorenc-Plucińska 2015). To our knowledge, there are no proteomic reports on aquatic plant-chromium interactions except that of Bah et al. (2010) which brings comparative data on Cr, Cd, and Pb influence on *Typha angustifolia* L. protein profiles. For the above reasons, the presented work is the first one to provide evidence on chromate stress-induced proteome alterations in a macrophyte with entirely submerged assimilative organs that is *C. cophocarpa* Sendtn. The expected result of this study is to find important protein changes upon chromate treatment, which we believe could explain the unusual capabilities of chromium phytoremediation by *C. cophocarpa*.

Our earlier research work revealed that Cr(VI) presence negatively affected *C. cophocarpa* shoots leading to several physiological changes such as a decrease of photosynthetic pigment content and a decline of photosynthesis quantum efficiency, distortion of leaf structure, and electrolyte leakage (Augustynowicz et al. 2010, 2013a,b). Moreover, chromate uptake was followed by its rapid reduction (Augustynowicz et al. 2013a, 2016) similarly to the other tested macrophytes *Eichhornia crassipes* (Mart.) Solms, *Salvinia auriculata* Aubl., *Pistia stratiotes* L., and *Spirodela polyrhiza* (L.) Schleid. (Espinoza-Quiñones et al. 2009; Kaszycki et al. 2005).

Unexpectedly, the results of 1D SDS-PAGE protein analyses indicated no visible alterations in the proteome of *C. cophocarpa* incubated with Cr(VI). Therefore, a more detailed study was undertaken employing two-dimensional electrophoresis, which enabled higher resolution of protein mapping as based on pI and *M*_W_ parameters. 2D electrophoreses yielded significant differences in the resultant protein profiles and showed changes in accumulation of several proteins as exemplified in Fig. 3. It should be emphasized, however, that many of the protein changes observed for a particular physiological experiment might not be linked directly to the chromate stress but rather to indigenous proteome pattern variability, which is typical of 2DE-based profiling. This variability may result from the noisiness of gene expression (Chalancon et al. 2012), intrinsic physicochemical diversity of proteins, and protein abundance differences between individual plant objects (Chandramouli and Qian 2009; Jorrín-Novo et al. 2009; Lopez 2007). The above reasons made us pick out only these spots whose densities changed in all of the gel pair replicates. Such an approach involving stringent conditions for selecting differentially abundant proteins enabled us to detect five protein alterations qualified as chromate stress specific for *C. cophocarpa* treated with 1-mM Cr(VI) for 72 h (Table 1).

In proteomic studies, the number of differentiating proteins in plants exposed to environmental stressors may vary in a broad range of several to several hundred detected spots (Kosová et al. 2011). In our recently launched experiments on 72-h chromate treatment of three other tested submerged macrophytes (*S. polyrhiza*, *Elodea canadensis* Michx., and *Lemna trisulca* L.), we revealed the total of 13, 12, and 21 differentially abundant proteins, respectively (Kaszycki et al., manuscript in preparation). It should be pointed out here that, besides methodological constraints of 2DE (DalCorso et al. 2013; Jorrín-Novo et al. 2009; Lopez 2007; Visioli and Marmiroli 2013), the resultant protein profile of abiotically stressed plant strongly depends on the time scale of observation. Some of the early-response changes including oxidative stress reactions can be observed in a matter of hours (Kosová et al. 2011; Singh et al. 2013), whereas enhanced proteomic rearrangements are typically best pronounced within 3–5 days, which is the time required for a plant to evolve an altered phenotype due to gene induction and protein biosynthesis (Forni et al. 2012; Kosová et al. 2011; Zhao et al. 2005). In turn, in longer-term experiments, mechanisms of plant acclimation and adaptation to harsh conditions (see Bah et al. 2010 for an example) may lead to further differences in proteomic profiles.

The proteomic changes observed in this study (see Table 1) can account for several important aspects of physiologically altered phenotype of *C. cophocarpa* under chromate stress. The case of protein spot 1 (a putative FQR1 enzyme) is discussed below in a more detail since it has possible direct consequences in terms of chromate resistance and bioremediation. The light harvesting chlorophyll *a*/*b* binding protein (LHCB), identified in spot 2, was strongly induced by Cr(VI). This is an abundant membrane apoprotein of photosystem II (PSII) and is normally complexed with chlorophyll and xanthophylls to serve as the antenna complex (Jansson 1999). Importantly, the expression of the *LHCB* gene was found to depend upon environmental conditions including, among others, oxidative stress (Xu et al. 2012). Therefore, the induction of this protein suggests its role in Cr bioremediation; however, at this stage, it is difficult to propose a particular mechanism. So far, LHCB proteins were found to be either up-regulated by chromate in a Cr(VI)-tolerant canola (*Brassica napus* L.) cultivar (Yildiz and Terzi 2016) or inhibited in a Cr(VI)-hypersensitive microphyte *Pseudokirchneriella subcapitata* (Korshikov) F. Hindák (Vannini et al. 2009).

RuBisCO (ribulose bisphosphate carboxylase), the enzyme catalyzing a key reaction of carbon dioxide fixation in photosynthesis, was down-regulated (spots 3 and 4). This was an expected result since chromate at 1-mM concentration was shown to inhibit photosynthesis of *C. cophocarpa* (Augustynowicz et al. 2010, 2013b). RuBisCO down-regulation was shown in many plant models and can be regarded as a typical response to abiotic stress including heavy metal treatments (Ahsan et al. 2009; Kosová et al. 2011). Accordingly and similarly to our observations, its accumulation decreased upon 3-day chromate incubation of both canola (Yildiz and Terzi 2016) and *P. subcapitata* (Vannini et al. 2009); however, under conditions of long-term (30-day) adaptation to Cr(VI), the enzyme was significantly up-regulated in *Typha angustifolia* (Bah et al. 2010).

Since Cr(VI) treatment is typically associated with the oxidative stress caused by elevated levels of ROS (see “Introduction”), it was of interest to verify whether *C. cophocarpa* triggered any relevant enzymatic radical-scavenging mechanisms and whether it could adapt during a prolonged (3-day) incubation with Cr(VI). The activities of three selected antioxidant enzymes were assayed, i.e., SOD (EC 1.15.1.1), CAT (EC 1.11.1.6), and (a sum of) peroxidases (Px). These enzymes are known as oxidative stress markers in plants (Geebelen et al. 2002) and were reported earlier to be responsive to chromate (Shanker et al. 2005). SOD acts as a scavenger of the superoxide anion, whereas CAT and Pxs catalytically eliminate hydrogen peroxide. At first, a 3-h treatment was examined, which revealed direct reaction to Cr(VI) by strongly increasing CAT and peroxidase activities. This early-response stage (an alarm phase of plant response as indicated by Kosová et al. 2011) is consistent with our previous observations which showed immediate reaction of *C. cophocarpa* shoots treated with chromate and manifested by a rapid (in a matter of hours) reduction of Cr(VI) to the Cr(V) intermediate which is generated upon Cr(VI) → Cr(III) bioconversion (Augustynowicz et al. 2013a, 2016). During prolonged (3-day) incubation, only Px activities were kept elevated while CAT was back at the level determined for control plants.

SOD, CAT, and peroxidase induction and/or activation are known to play a major role in heavy metal detoxication in plants (Ahsan et al. 2009). However, in plants treated with chromate, as reviewed by Shanker et al. (2005) and Singh et al. (2013), the activity of these enzymes increased only at relatively low chromate concentrations (usually of the order of micromoles per liter) whereas the excess of Cr(VI) (most typically above 100 μM) led to significant activity inhibition. The fact that in *C. cophocarpa* the activity of both SOD and CAT remained at control levels under harsh conditions of 3-day 1-mM Cr(VI) presence, it brings additional evidence that the studied macrophyte could induce enhanced resistance to oxidative stress. In the context of the above, the mechanism of long-term activation of peroxidases under chromate treatment needs further research. These enzymes were previously shown to be efficient scavengers of heavy metal borne H_2_O_2_ (Ahsan et al. 2009). However, the increase of Px activities as observed in enzymatic assays was not reflected in 2DE analyses by elevated abundance of any known peroxidase. This result can be explained by the stimulation of enzymatic activity with biochemical regulatory mechanisms rather than by induced expression. Similar discrepancies were reported by other authors who detected peroxidase activity stimulation by Cr (Zemleduch-Barylska and Lorenc-Plucińska 2015) or Cd (e.g., Yang et al. 2015; Kieffer et al. 2009) but observed no accumulation changes in 2DE profiling.

Taken the above findings together, it appears that *C. cophocarpa* shoots can adapt to 1-mM Cr(VI) within 3 days by increasing activity of peroxidases while maintaining the levels of the other antioxidant enzymes: SOD and CAT. This, in turn, indicates the tendency to suppress oxidative stress and suggests that chromate presence can launch protective mechanisms resulting in lowered pool of toxic ROS.

The FQR1 protein as identified in spot 1 of the electrophoretic gel (the highest score of *V. vinifera* NAD(P)H quinone dehydrogenase, see Fig. 3 and Table 1) was shown to be very strongly and specifically induced by chromate. Its presence was always well pronounced only in Cr-treated plants whereas both under control conditions and in all cases of other stressors (Fig. 4a, c–f), the electrophoretic gels lacked accumulation of any protein related to FQR1. A closer look at this enzyme enables us to suggest its direct involvement in chromate bioremediation as a putative quinone and/or chromate reductase. Extensive database search (www.ebi.ac.uk/interpro/protein/Q9LSQ5, accession date: July 2017) following the NCBI protein identification accessions gave more information on the enzyme identity. The FQR1 domain is not unique and can be found in several flavoproteins. It belongs to a big family of highly conserved flavin-binding reductases occurring in plants, fungi, archaea, and eubacteria and shares some homologies with mammalian quinone reductases (Laskowski et al. 2002). In particular, a NAD(P)H:quinone oxidoreductase can reduce quinones to the hydroquinone state in a two-electron reaction, which prevents the interaction of the semiquinone with O_2_ and thus disables production of toxic superoxide. So, the enzyme has been postulated to function as a protective agent in oxidative stress response and to participate in detoxifying reactions (Berczi and Moller 2000; Laskowski et al. 2002).

Zymographic analyses (see the enzymatic band indicated by an arrow in Fig. 5) made it possible to prove that the QR activity was indeed induced in *Callitriche* treated with chromate. To detect an in vitro QR activity in Cr(VI)-stressed plants, we used a modified assay enabling to visualize the enzyme in native-PAGE gels. Such an approach was necessary since the standard laboratory assays (data unshown) failed to bring conclusive results, yielding high apparent (background) activities, possibly due to high abundance of phenolics in *C. cophocarpa* (Augustynowicz et al. 2014a).

It should be emphasized here that QR activities in Cr(VI)-stressed plants have not been reported to date. Although quinone oxidoreductases are ubiquitous among plant species, there are only a few proteomic studies providing evidence of QRs being responsive to other heavy metals or metalloids. First, Requejo and Tena (2005) showed a p-benzoquinone reductase (bQR) induction in a maize (*Zea mays* L.) root proteome treated with arsenic and proposed a protective role of the enzyme against oxidative stress. For cadmium-stressed plants, Kieffer et al. (2009) reported strong up-regulation of FQR1 as well as several other bQR-like and putative QR proteins in poplar leaves. Zhao et al. (2011), in turn, revealed enhanced accumulation of bQR in a leaf proteome of a Cd-hyperaccumulator *Phytolacca americana* L. Recently, Chen et al. (2015) detected a protein described as a “putative quinone reductase 2” in the proteome of Cu-stressed rice (*Oryza sativa* L.).

There is scientific evidence enabling us to hypothesize that the induction of the newly identified FQR1 enzyme plays an important role in the mechanism of chromate bioreduction as observed in *Callitriche*. Cr(VI) biological reduction process has been documented for plants as well as for all other organisms: pro- and eukaryotic microbes, fungi, algae, and animals (Joutey et al. 2015; Singh et al. 2013; Zhitkovich 2011). In general, hexavalent chromate reduction may involve both extra- and intracellular processes and both enzymatic and non-enzymatic mechanisms. For Cr(VI) bioconversion inside cell, the reduction process may have two contradictory aspects: first, it can result in cellular toxicity due to oxidative stress, and second, it can serve as a bioremediation (resistance) mechanism. In the first case, single-electron reactions lead to generation of detrimental ROS (Cheung and Gu, 2007; Zhitkovich 2011; Thatoi et al. 2014) which in turn induce antioxidative metabolism producing cellular antioxidants (Panda and Choudhury 2005; Singh et al. 2013). In the second case, the cellular Cr(VI) → Cr(III) conversion is achieved preferably via a two-electron transfer systems which enable an organism to cope with the chromate stress by safely (that is minimizing ROS production) reducing the original hexavalent form to the less mobile and thus less reactive Cr(III) (Ramírez-Díaz et al. 2008; Joutey et al. 2015).

Several enzymes capable of Cr(VI) reduction have been described, first identified in bacteria where various chromate reductases were shown to influence microbial resistance to Cr(VI) (Ramírez-Díaz et al. 2008; Thatoi et al. 2014 and the references therein). Note that these reported chromate-reducing activities relied upon either one- or two-electron mechanisms, which resulted in different strain sensitivities to chromate as discussed above. Importantly, the two-electron Cr(VI) reduction systems that enable safer Cr(VI) detoxication were typically linked to soluble flavin mononucleotide-binding oxidoreductases producing QR activities. These enzymes were induced by chromate and identified as bacterial chromate reductases because they could use Cr(VI) as a possible terminal electron acceptor (Eswaramoorthy et al. 2012; Thatoi et al. 2014).

Besides bacteria, Cr(VI)-reducing activities were also observed in yeast, microalgae, fungi (Joutey et al. 2015; Viti et al. 2014), as well as plants (Prado et al. 2016; Shanker et al. 2009) including macrophytes (Espinoza-Quiñones et al. 2009; Kaszycki et al. 2005) and *C. cophocarpa* in particular (Augustynowicz et al. 2013a, 2016). Importantly, for the latter case, chromate bioreduction occurred exclusively within the plant shoots and was not observed outside the macrophyte tissue (that is in a surrounding growth medium). This result was based on a low-frequency in vivo electron paramagnetic resonance study (Augustynowicz et al. 2013a) which revealed that *C. cophocarpa* chromate phytoremediation involved Cr(VI) intratissular reductive conversion generating a Cr(V) intermediate.

As regards plants, chromate reductases have not been identified in vivo, yet. However, in a study on *Arabidopsis thaliana* (L.) Heynh., Sparla et al. (2003) documented that a flavoenyzme NAD(P)H:quinone oxidoreductase (NQR) shared sequence homologies with some bacterial chromate reductases. This led the authors to the idea that the enzyme could function as a chromate reductase and thus promote Cr phytoremediation. Using *A. thaliana* homogenates, they detected a NQR-based NADPH-chromate reductase activity in in vitro tests. Note however, that the *Arabidopsis* NQR protein, unlike the enzyme identified in this study, was not inducible and it belonged to a different family of quinone reductases, poorly related to the FQR domains (Heyno et al. 2013). Later, Shanker et al. (2009), based on extensive bioinformatic database search, speculated that putative plant chromate reductases homologous to the microbial ones might take part in Cr(VI) detoxification.

Taking all the above facts into consideration it is tempting to suggest that *C. cophocarpa* FQR1 quinone reductase shows chromate-reducing activity and is an important factor involved in Cr(VI) bioremediation. However, the postulated role of the enzyme still awaits direct experimental evidence. If the novel enzyme proved to reduce Cr(VI) substrate both in vitro and in vivo, its induction with chromate would indicate that *C. cophocarpa* is the first known aquatic plant to have developed some specific enzymatic detoxifying mechanism against Cr(VI) action. This, in turn, would imply that our plant model could combine high Cr-resistance with enhanced metal accumulation and biomass yield thus making *C. cophocarpa* even better choice for efficient phytoremediation of polluted waters.

## Concluding remarks

Proteomic pattern alterations observed for *C. cophocarpa* treated with sublethal chromate levels are not extensive and involve only several proteins revealing changed accumulation. Among the differentially abundant proteins, the induction of a flavoenzyme FQR1-like quinone reductase (QR) is the most significant finding. This enzyme is expected to be a detoxification factor that protects the cells against the Cr(VI)-generated oxidative stress via catalyzing transfer of two electrons from NAD(P)H to hexavalent chromium. QR expression is chromate-specific since it is not induced by any other stressful conditions (salt, temperature, and oxidative stress) and in *C. cophocarpa*, it might function as a part of the evolved enzymatic Cr(VI) reduction pathway, analogous to that described for aerobically grown Gram-negative rods.

This is the first study to show specific plant response to chromate stress by inducing an antioxidant enzyme with potential chromate reductase activity. For the case of *C. cophocarpa* and possibly other macrophytes capable of efficient Cr phytoremediation, the existence of such a mechanism would contribute to their chromium-resistance phenotype and thus enhance environmental applicability.

## Electronic supplementary material

Supplementary Figures Electrophoretic data obtained upon independent physiological repetitions of *Callitriche cophocarpa* treated with 1-mM chromate. (DOCX 3891 kb)

Supplementary Table 1 MS/MS identification data for differentially abundant proteins of *Callitriche cophocarpa* treated with 1-mM chromate. (XLSX 41 kb)
