# Supplementary material for: Callitriche cophocarpa (water starwort) proteome under chromate stress: evidence for induction of a quinone reductase
Source: Environ Sci Pollut Res Int. 2018 Jan 13;25(9):8928–42. doi: 10.1007/s11356-017-1067-y (PMC5854755; doi:10.1007/s11356-017-1067-y)
Supplement: Supplementary file 1 — Electrophoretic data obtained upon independent physiological repetitions of Callitriche cophocarpa treated with 1-mM chromate. (DOCX 3891 kb) [file 11356_2017_1067_MOESM1_ESM.docx]

Supplementary Fig. 1

SDS-PAGE protein profiles of shoot extracts of *Callitriche cophocarpa* treated with 1 mM chromate for 72 h (Cr).
C*,* control (untreated plant); ST, protein standard markers.

Letters (a), (b), (c) indicate the individual gels obtained upon three independent physiological experiments

**
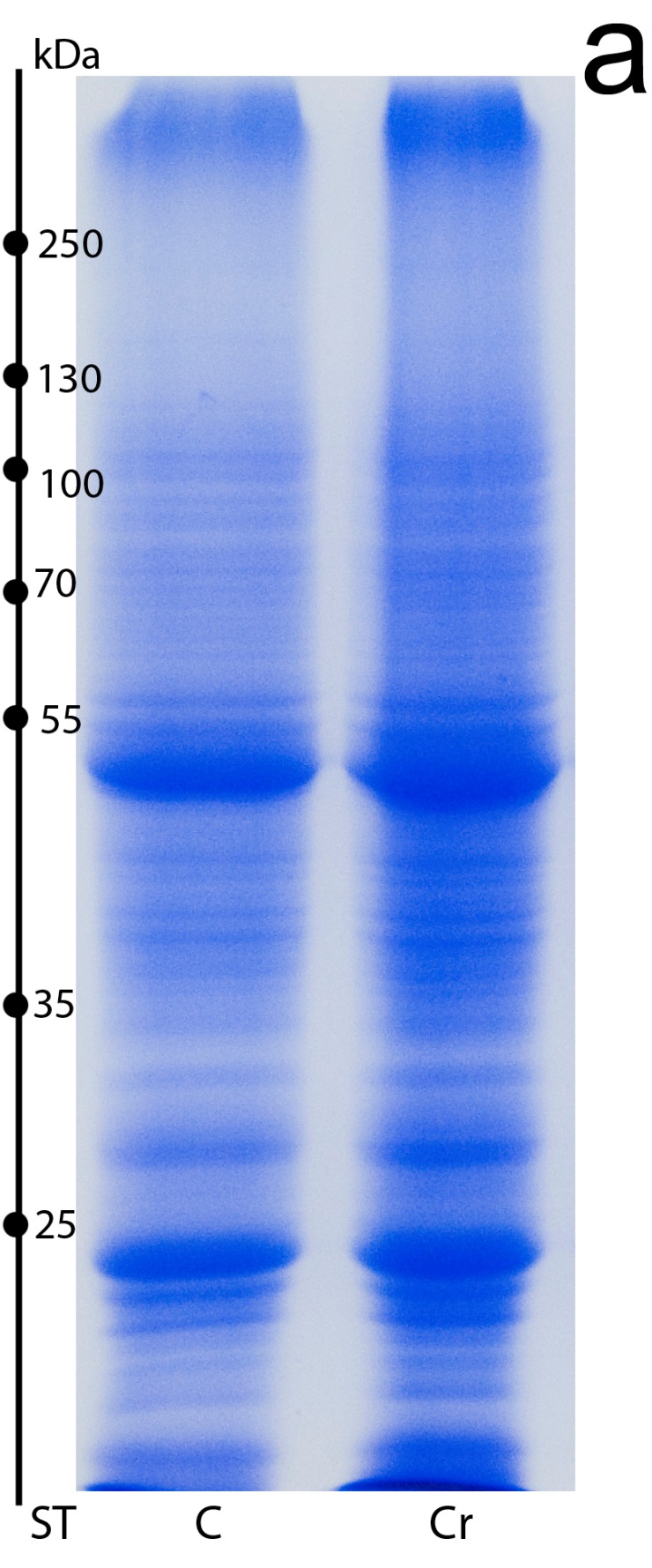
**

**
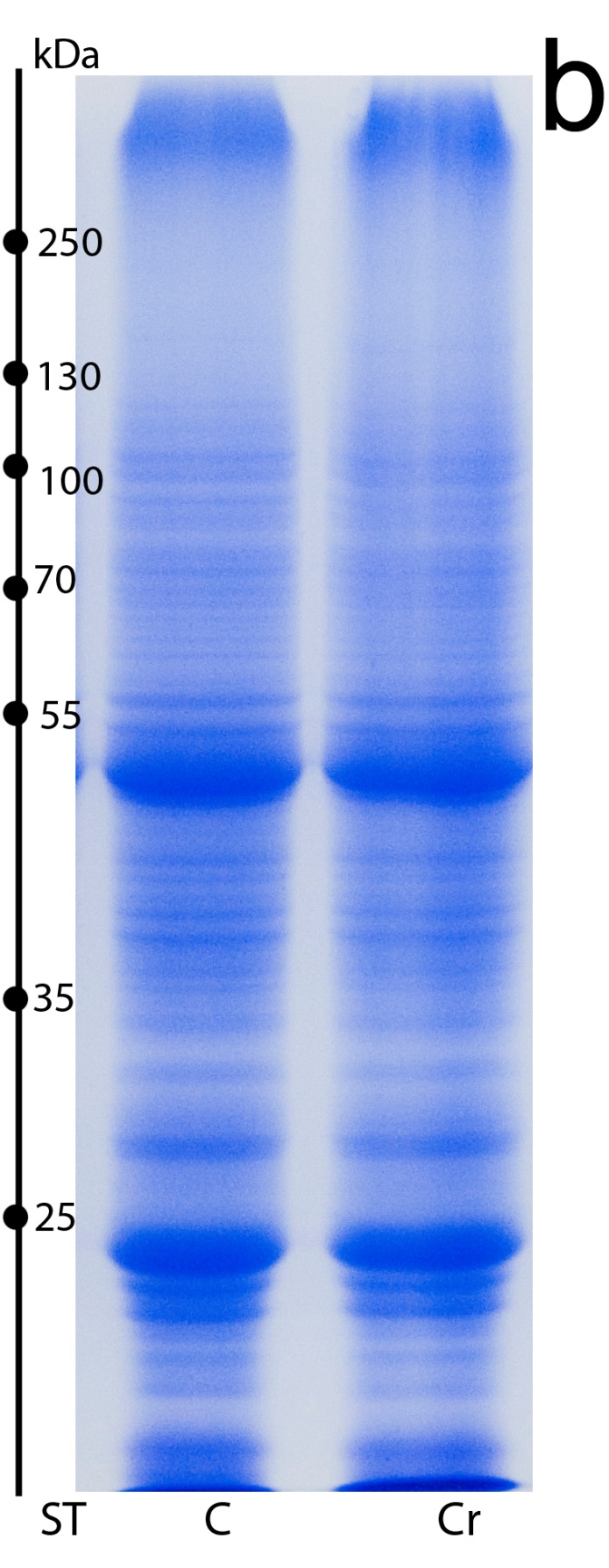
**

**
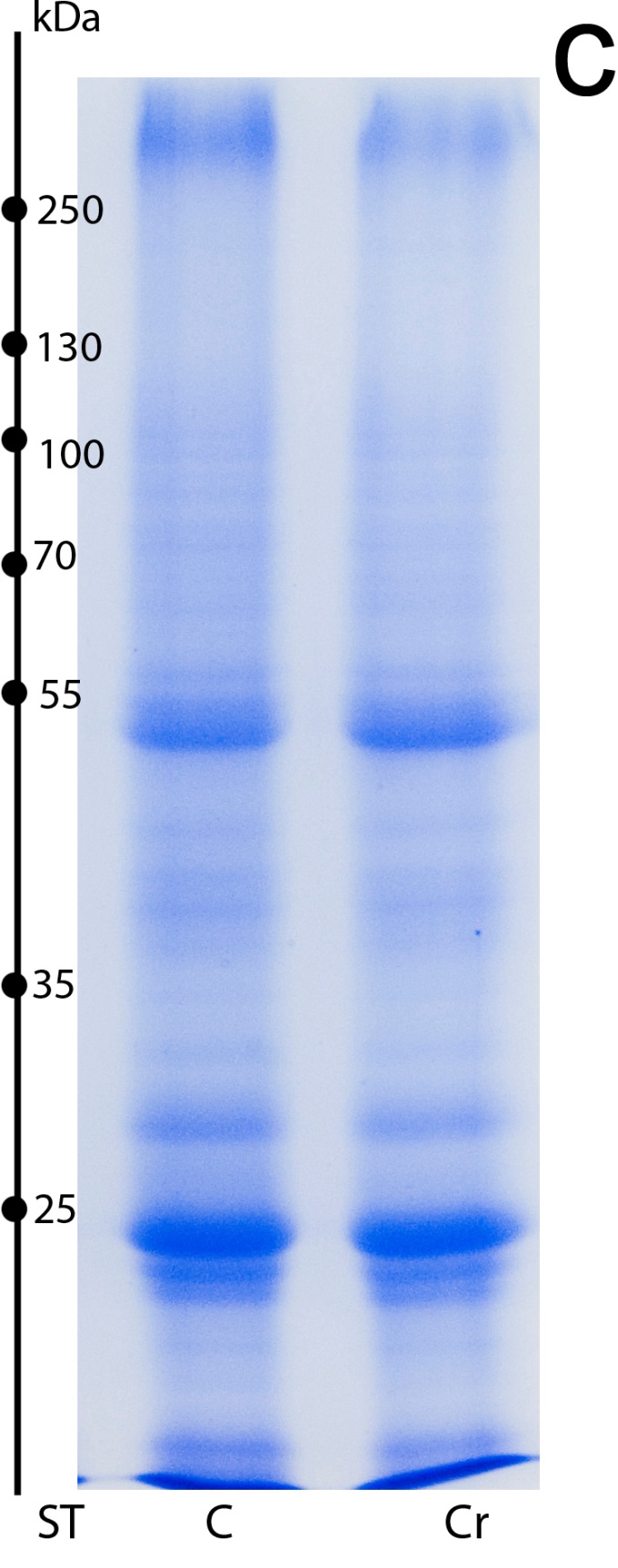
**

Supplementary Fig. 2

Two-dimensional (2DE) proteome mapping of *C. cophocarpa* shoots after treatment with 1 mM potassium chromate
for 72 h (gel 2, right) as compared to the untreated controls (gel 1, left). The isoelectric focusing (IEF) step was performed at pI range 3-10. The differentiating spots are numbered consecutively and marked with circles; spots no. 1, 2 represent the Cr(VI)-induced proteins (green circles), spots no. 3, 4, 5 indicate the down-regulated proteins (red circles).

Letters (a), (b), (c) indicate the individual gels obtained upon three independent physiological experiments


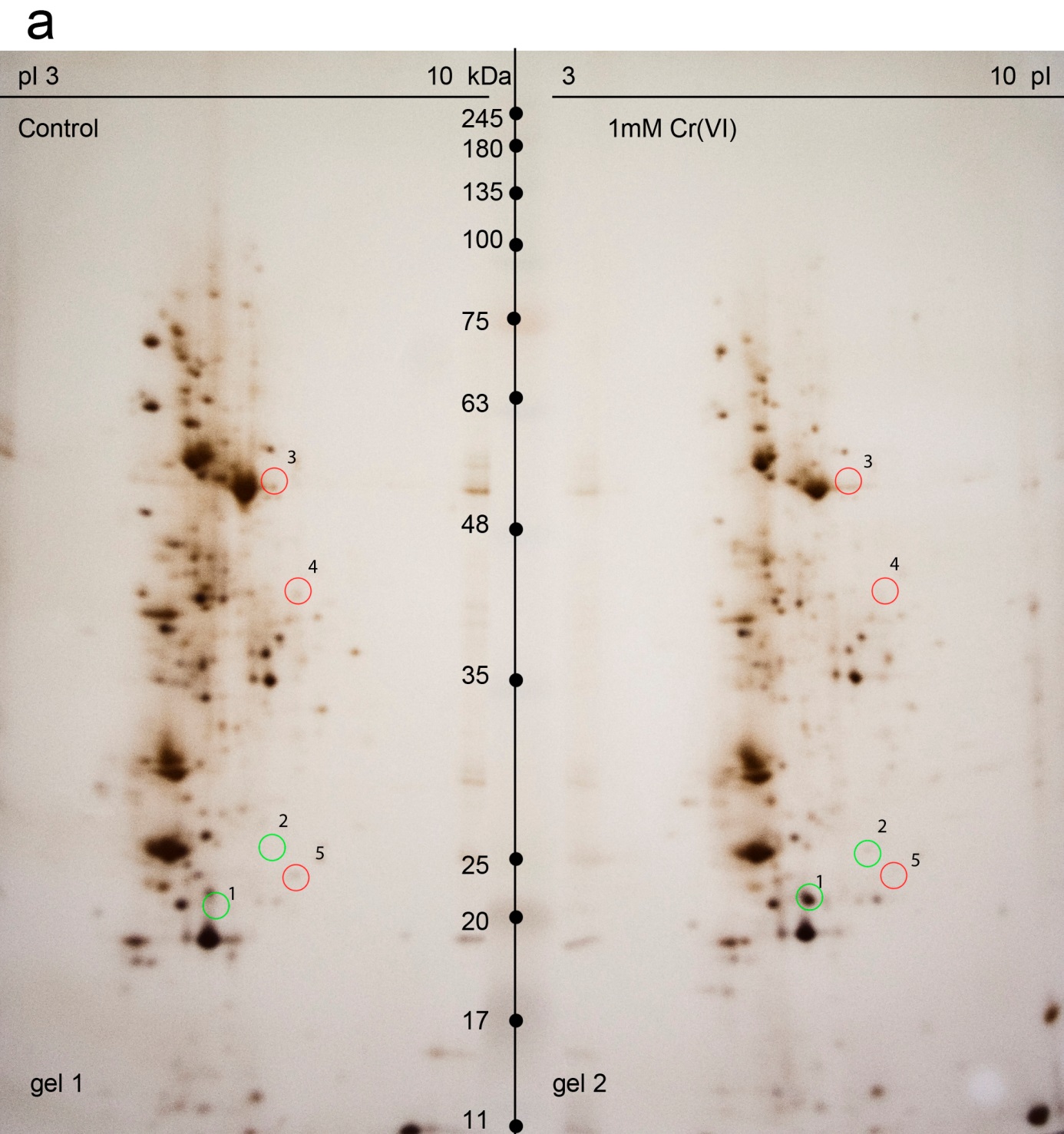


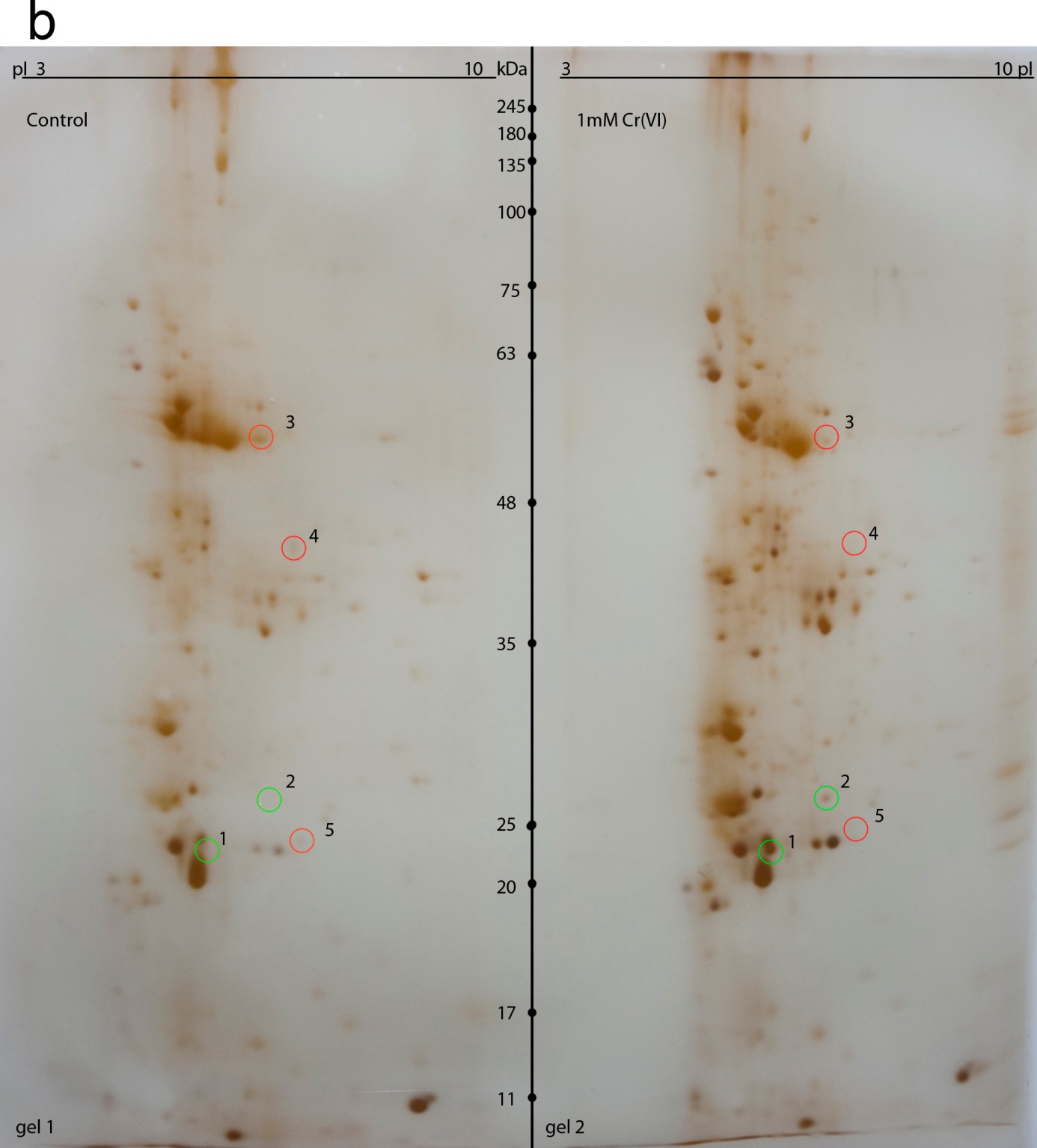


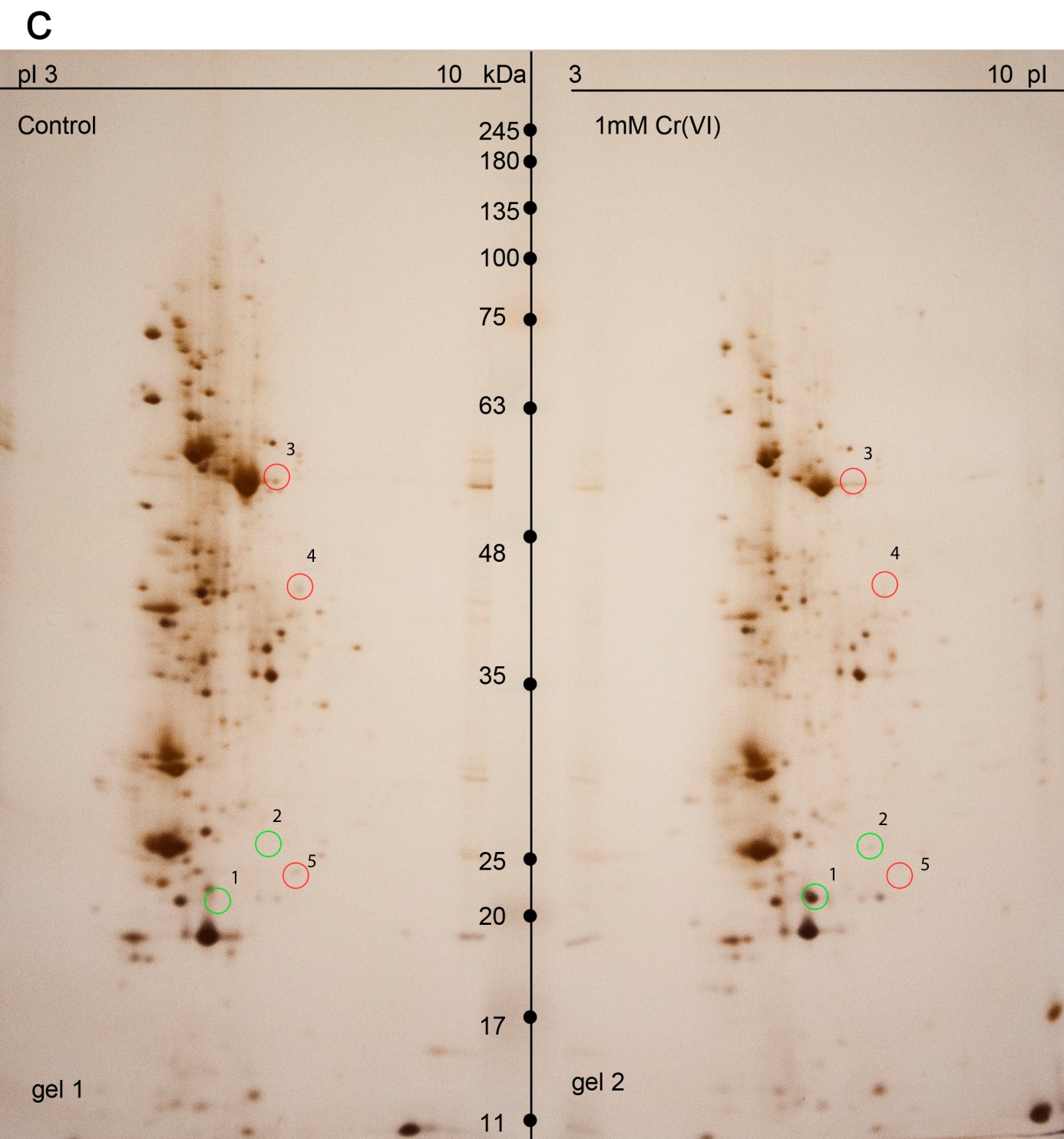


Supplementary Fig. 3

Two-dimensional (2DE) proteome mapping of *C. cophocarpa* shoots after treatment with 1 mM potassium chromate
for 72 h (gel 2, right) as compared to the untreated controls (gel 1, left). The isoelectric focusing (IEF) step was performed at pI range 5-8. The differentiating spots are numbered consecutively and marked with circles; spots no. 1, 2 represent the Cr(VI)-induced proteins (green circles), spots no. 3, 4, 5 indicate the down-regulated proteins (red circles).

Letters (a), (b), (c) indicate the individual gels obtained upon three independent physiological experiments


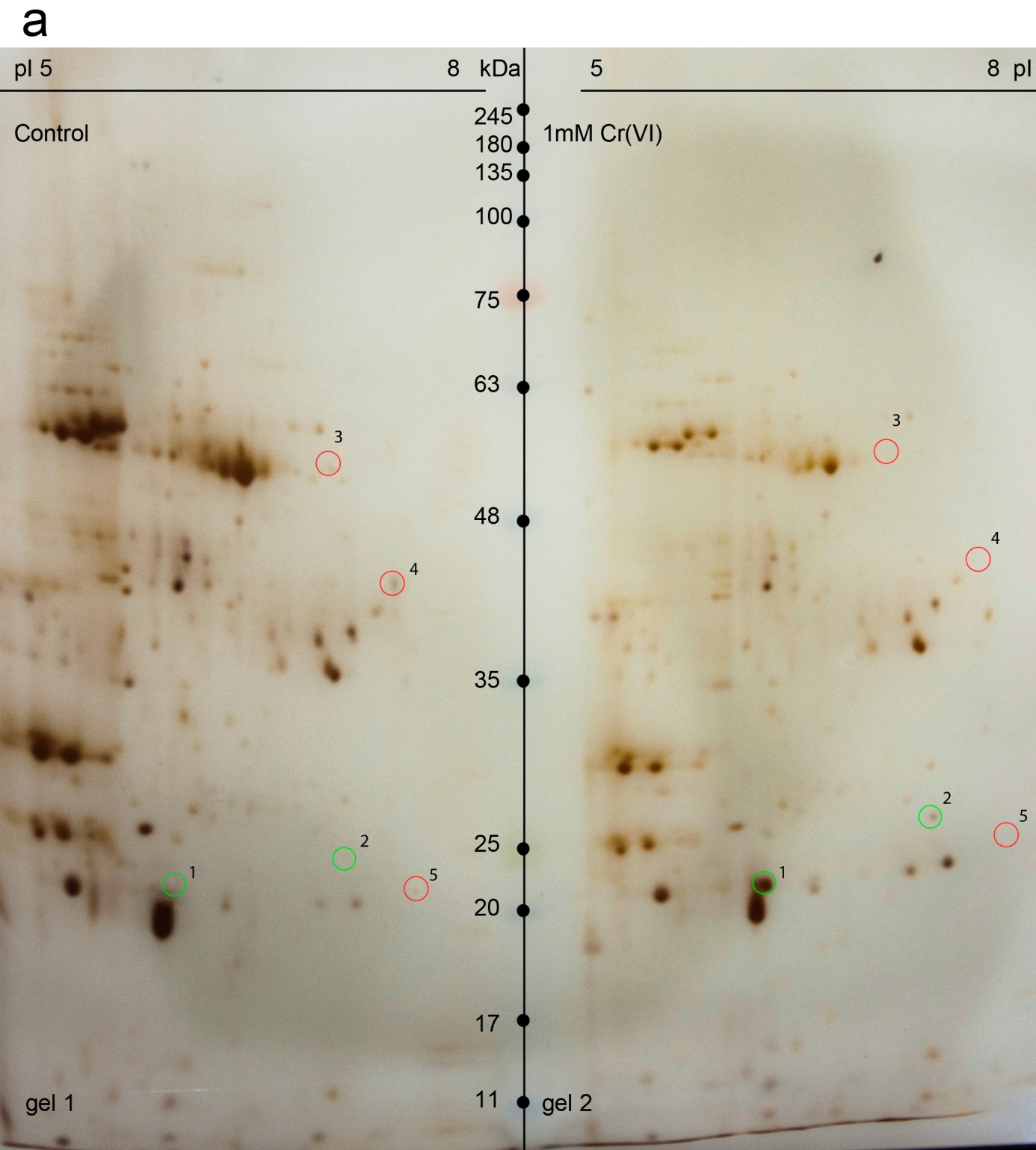


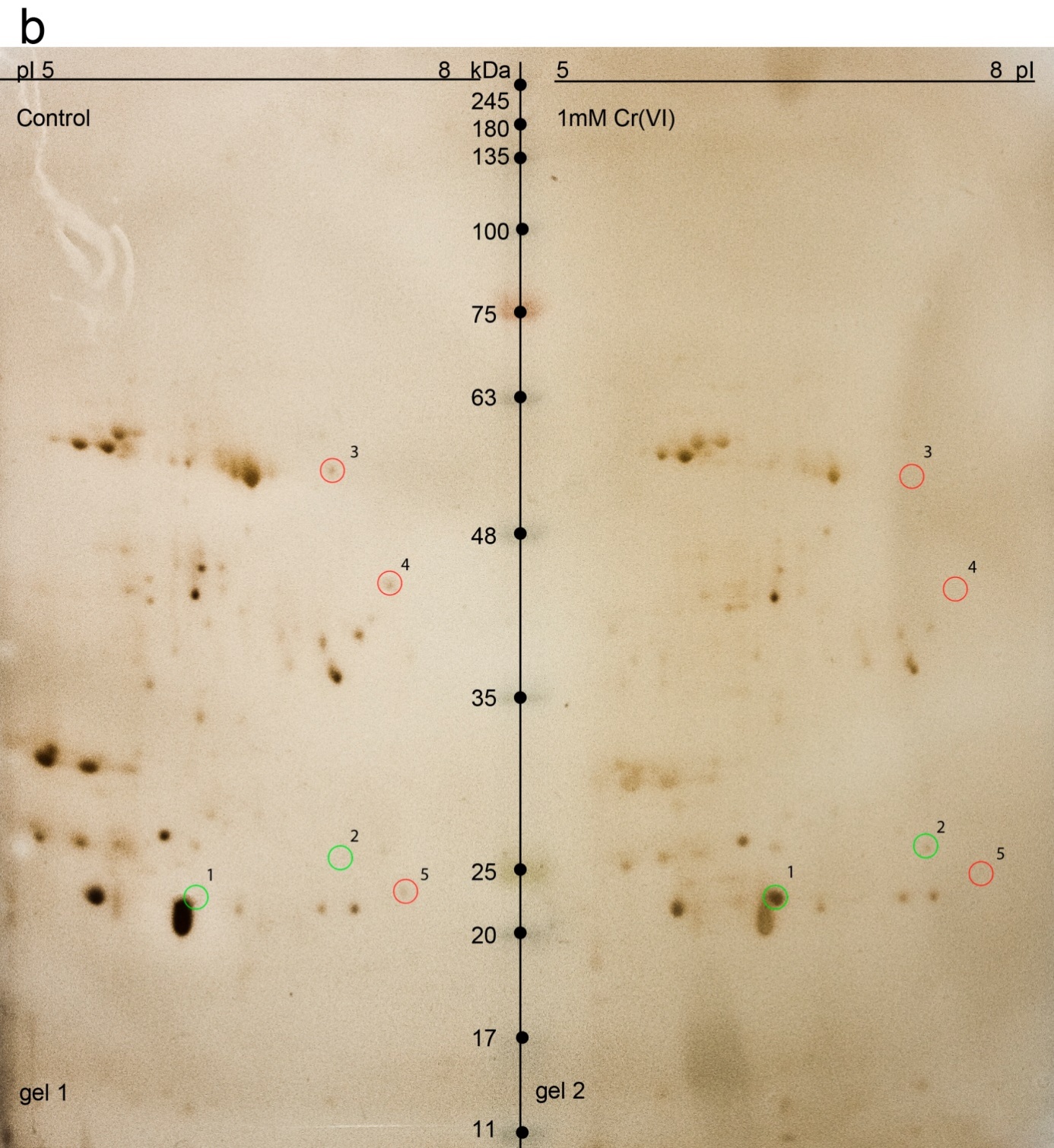


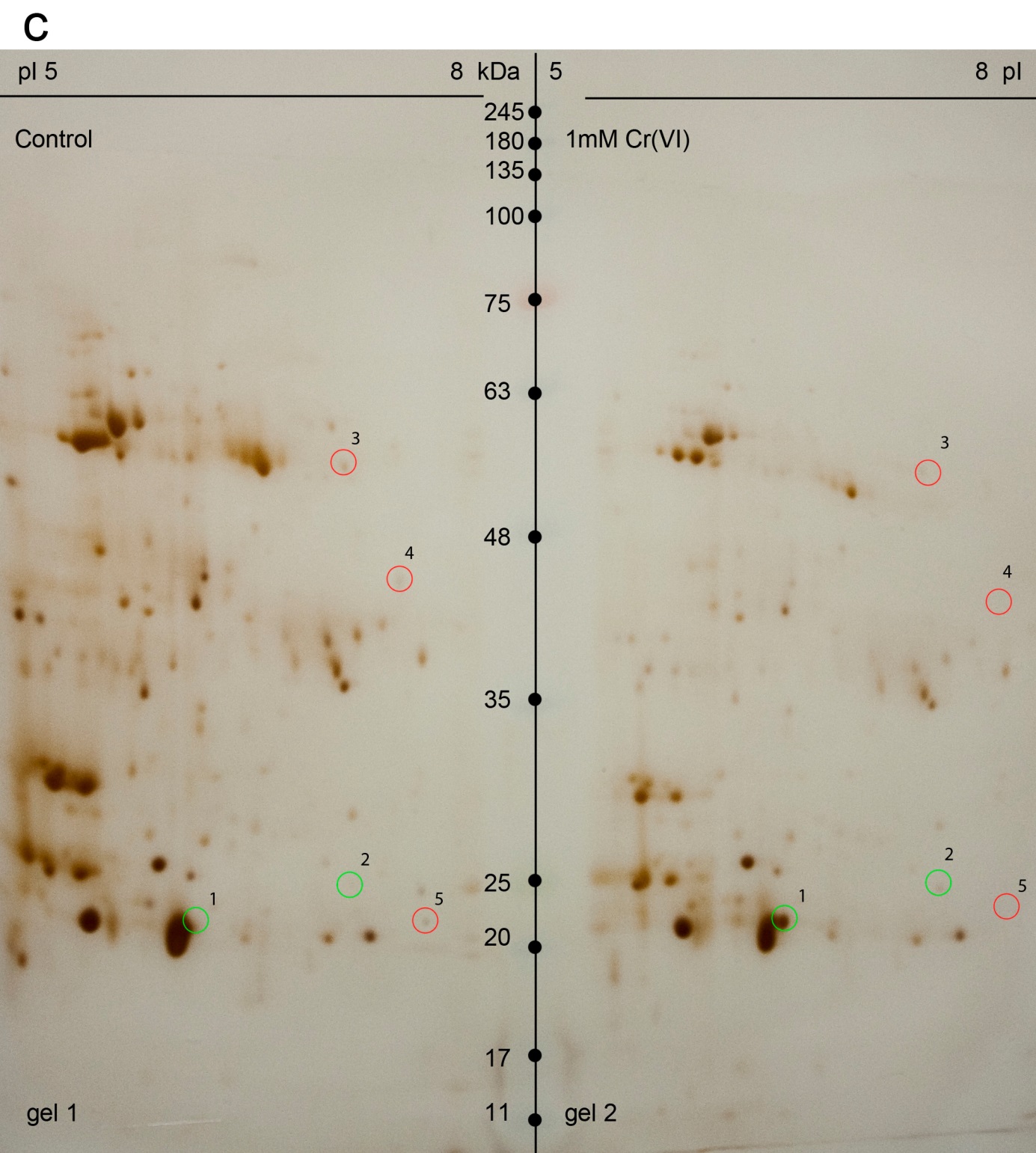


Supplementary Fig. 4

Zymographic analyses of shoot protein extracts of *Callitriche cophocarpa* treated with 1 mM Cr(VI) for 72 h. Enzymatic staining with a menadione substrate was done to reveal production of a novel quinone reductase activity as indicated by an arrow. C, control

Letters (a), (b), (c) indicate the individual zymograms obtained upon three independent physiological experiments


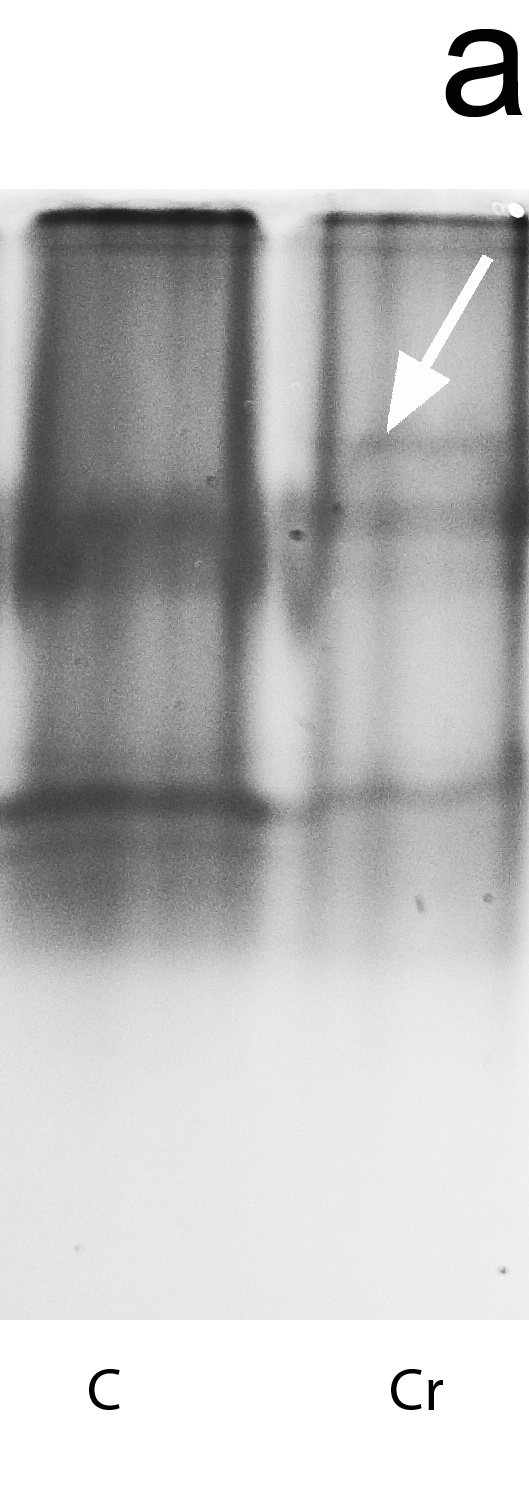


**
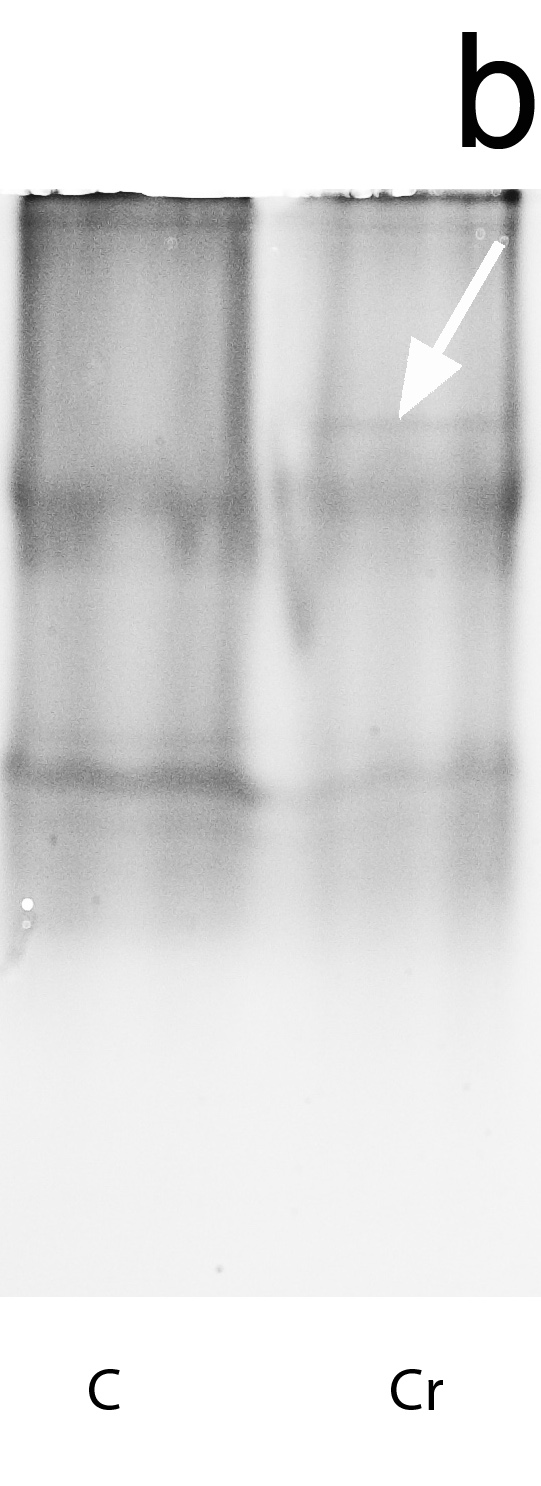
**

**
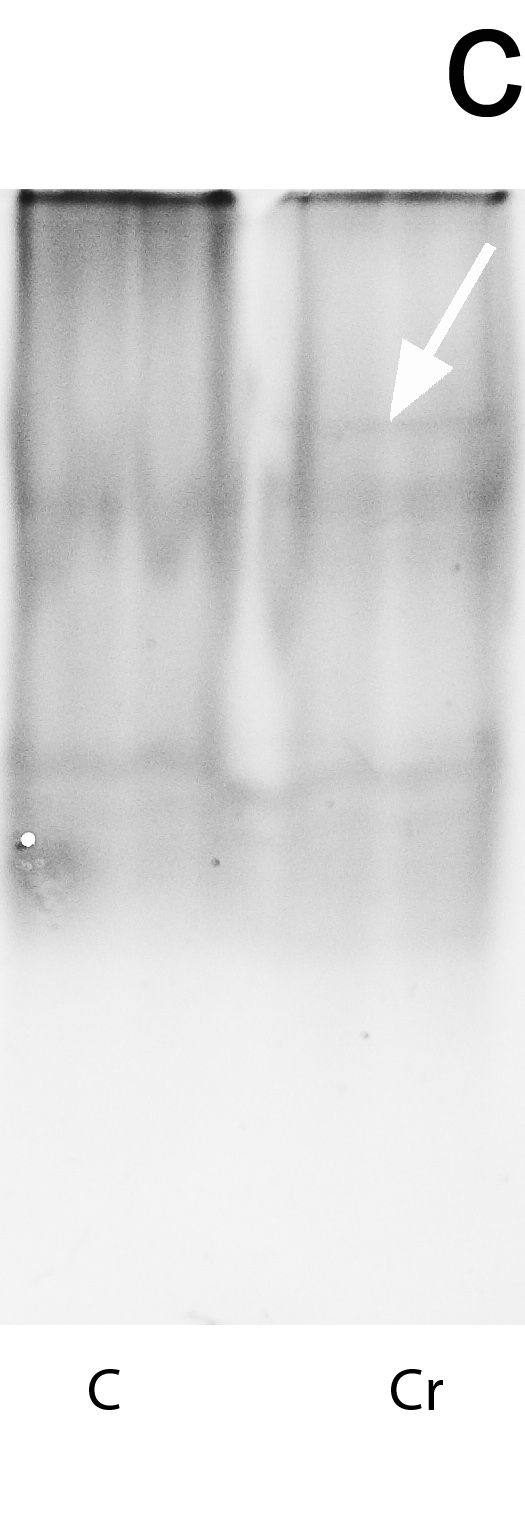
**
